# Supplementary material for: Locus specific human endogenous retroviruses reveal new lymphoma subtypes
Source: bioRxiv. 2023 Jun 8:2023.06.08.544208. Preprint. [Version 1] doi: 10.1101/2023.06.08.544208 (PMC10274920; doi:10.1101/2023.06.08.544208)
Supplement: Supplement 1 — Supplementary Figure 1: Unique and differentially expressed HERV loci in the B-HM dataset. A. Upset plot of the number of unique and shared HERVs upregulated in each B cell type (p < 0.001, log2fold change > 1.5). B. Upset plot of the number of unique and shared HERVs downregulated in each B cell type (p < 0.001, log2fold change > 1.5). C. Volcano plot of differentially expressed HERVs in all cell types versus DZ, D. all versus LZ, E. all versus MB, and F. all versus NB. Supplementary Figure 2: Unique and differentially expressed HERV loci in the B-AG dataset. A. Upset plot of the number of unique and shared HERVs upregulated in each B cell type (p < 0.001, log2fold change > 1.5). B. Upset plot of the number of unique and shared HERVs downregulated in each B cell type (p < 0.001, log2fold change > 1.5). C. Volcano plot of differentially expressed HERVs in all cell types versus DZ, D. all versus LZ, E. all versus MB, and F. all versus NB. Supplementary Figure 3: Plasmablasts and bone marrow plasma cells express distinct HERV profiles compared to GC B cells in the B-AG dataset. A. Volcano plot of differentially expressed HERVs in all cell types versus BMPC, B. all versus PB. C. Heatmap of the top 75 upregulated genes and HERVs in PB (p < 0.001, log2fold change > 1.5), and D. BMPC. Supplementary Figure 4: Key features differentiating B-AG B cell subsets based on feature selection with DESeq2 LRT, Boruta, and Lasso. A. UpsetR plot displaying the number of features selected by DESeq2 lowest likelihood ratio (LTR), the random forest classification with the Boruta algorithm, and the randomized least absolute shrinkage and selection operator (LASSO) regression, with 11 features being selected by all three methods. B. Rpart decision tree, displaying that HERVP71A_8q24.13 differentiates plasma cells (PB and BMPC) from the rest of the B cells. HERVL_2p12a differentiates DZ from the remaining cell types, while HUERSP2_6p22.3 differentiates LZ from MB and NB. C. Normalized count [file media-1.pdf]

Supp Fig. 1

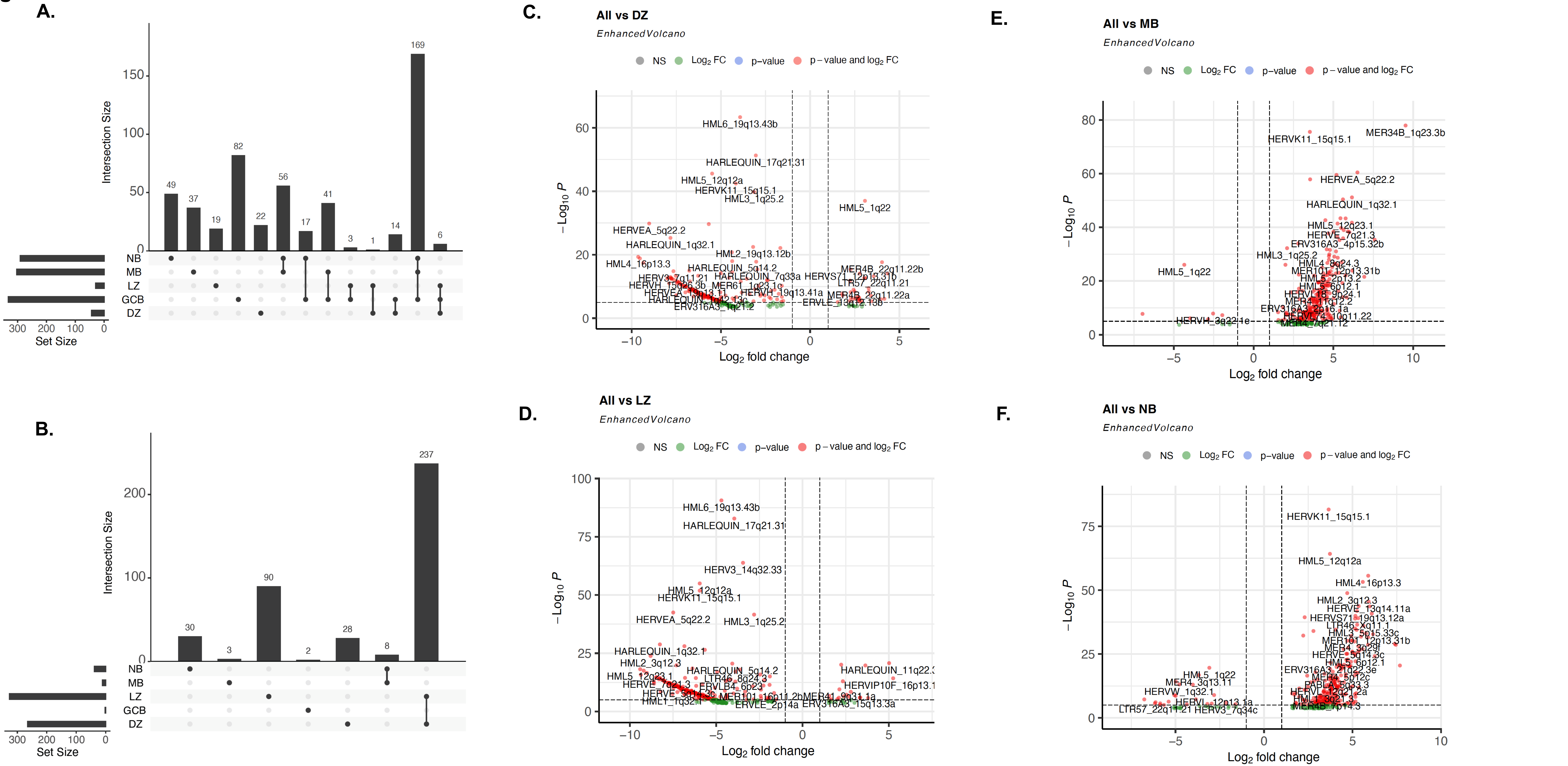

A.
Intersection Size
Set Size
NB
MB
LZ
GCB
DZ
49
37
19
82
22
56
17
41
3
1
14
169
6
B.
Intersection Size
Set Size
30
3
90
2
28
8
237
C.
All vs DZ
EnhancedVolcano
NS
Log2 FC
p−value
p − value and log2 FC
−Log10 P
Log2 fold change
HML6_19q13.43b
HARLEQUIN_17q21.31
HML5_12q12a
HERVK11_15q15.1
HML3_1q25.2
HML5_1q22
HERVEA_5q22.2
HARLEQUIN_1q32.1
HML2_19q13.12b
HML4_16p13.3
HARLEQUIN_5q14.2
MER4B_22q11.22b
D.
All vs LZ
EnhancedVolcano
NS
Log2 FC
p−value
p − value and log2 FC
−Log10 P
Log2 fold change
HML6_19q13.43b
HARLEQUIN_17q21.31
HERV3_14q32.33
HML5_12q12a
HERVK11_15q15.1
HERVEA_5q22.2
HML3_1q25.2
HARLEQUIN_1q32.1
HML2_3q12.3
HARLEQUIN_5q14.2
E.
All vs MB
EnhancedVolcano
NS
Log2 FC
p−value
p − value and log2 FC
−Log10 P
Log2 fold change
HERVK11_15q15.1
MER34B_1q23.3b
HERVEA_5q22.2
HARLEQUIN_1q32.1
HML5_1q22
F.
All vs NB
EnhancedVolcano
NS
Log2 FC
p−value
p − value and log2 FC
−Log10 P
Log2 fold change
HERVK11_15q15.1
HML5_12q12a
HML4_16p13.3
HML2_3q12.3

Supp Fig. 2

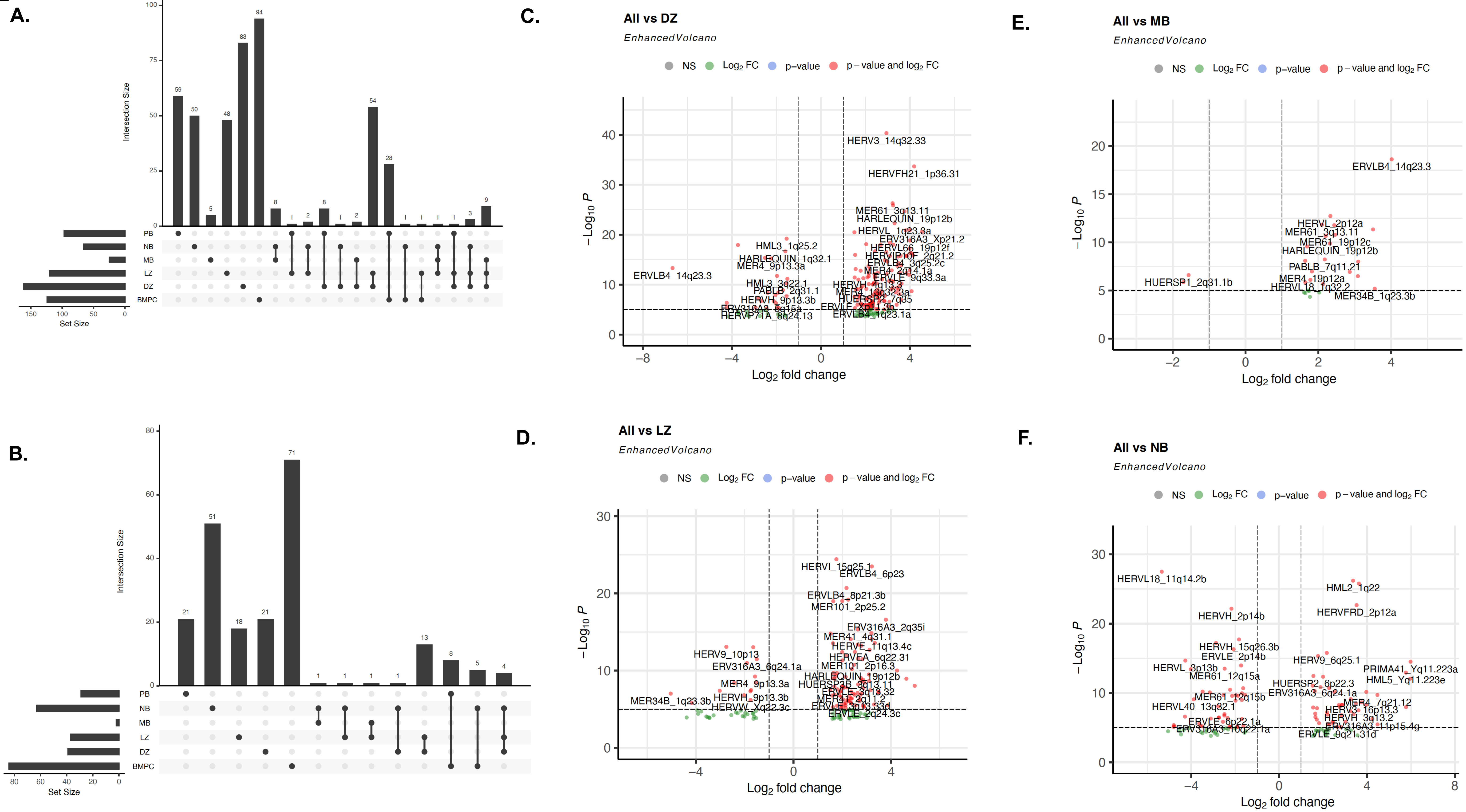
A.
Intersection Size
Set Size
PB
NB
MB
LZ
DZ
BMPC
B.
C.
All vs DZ
EnhancedVolcano
NS
Log2 FC
p–value
p – value and log2 FC
HERV3_14q32.33
HERVFH21_1p36.31
MER61_3q13.11
HARLEQUIN_19p12b
HERVL_1q23.3a
ERV316A3_Xp21.2
HERVL66_19p12f
HERVIP10F_2q21.2
ERVLB4_3q25.2c
MER4_2q14.1a
ERVLE_9q33.3a
HML3_1q25.2
HARLEQUIN_1q32.1
MER4_9p13.3a
ERVLB4_14q23.3
HML3_3q22.1
PABLB_2q31.1
HERVH_9p13.3b
ERV316A3_5q15a
HERVP71A_8q24.13
ERVLB4_1q23.1a
Log2 fold change
−Log10 P
D.
All vs LZ
HERVI_15q25.1
ERVLB4_6p23
ERVLB4_8p21.3b
MER101_2p25.2
ERV316A3_2q35i
MER41_4q31.1
HERVE_11q13.4c
HERVEA_6q22.31
MER101_2p16.3
HARLEQUIN_19p12b
HUERSP3B_3q13.11
HERV9_10p13
ERV316A3_6q24.1a
MER4_9p13.3a
HERVH_9p13.3b
MER34B_1q23.3b
HERVW_Xq22.3c
ERVLE_2q24.3c
E.
All vs MB
ERVLB4_14q23.3
HERVL_2p12a
MER61_3q13.11
MER61_19p12c
HARLEQUIN_19p12b
PABLB_7q11.21
MER4_19p12a
HERVL18_1q32.2
MER34B_1q23.3b
HUERSP1_2q31.1b
F.
All vs NB
HERVL18_11q14.2b
HML2_1q22
HERVFRD_2p12a
HERVH_2p14b
HERVH_15q26.3b
ERVLE_2p14b
HERV9_6q25.1
HERVL_3p13b
MER61_12q15a
PRIMA41_Yq11.223a
HML5_Yq11.223e
HUERSP2_6p22.3
ERV316A3_6q24.1a
MER61_12q15b
HERVL40_13q32.1
MER4_7q21.12
HERV3_16p13.3
HERVH_3q13.2
ERVLE_6p22.1a
ERV316A3_10q22.1a
ERV316A3_11p15.4g
ERVLE_9q21.31d

Supp Fig. 3

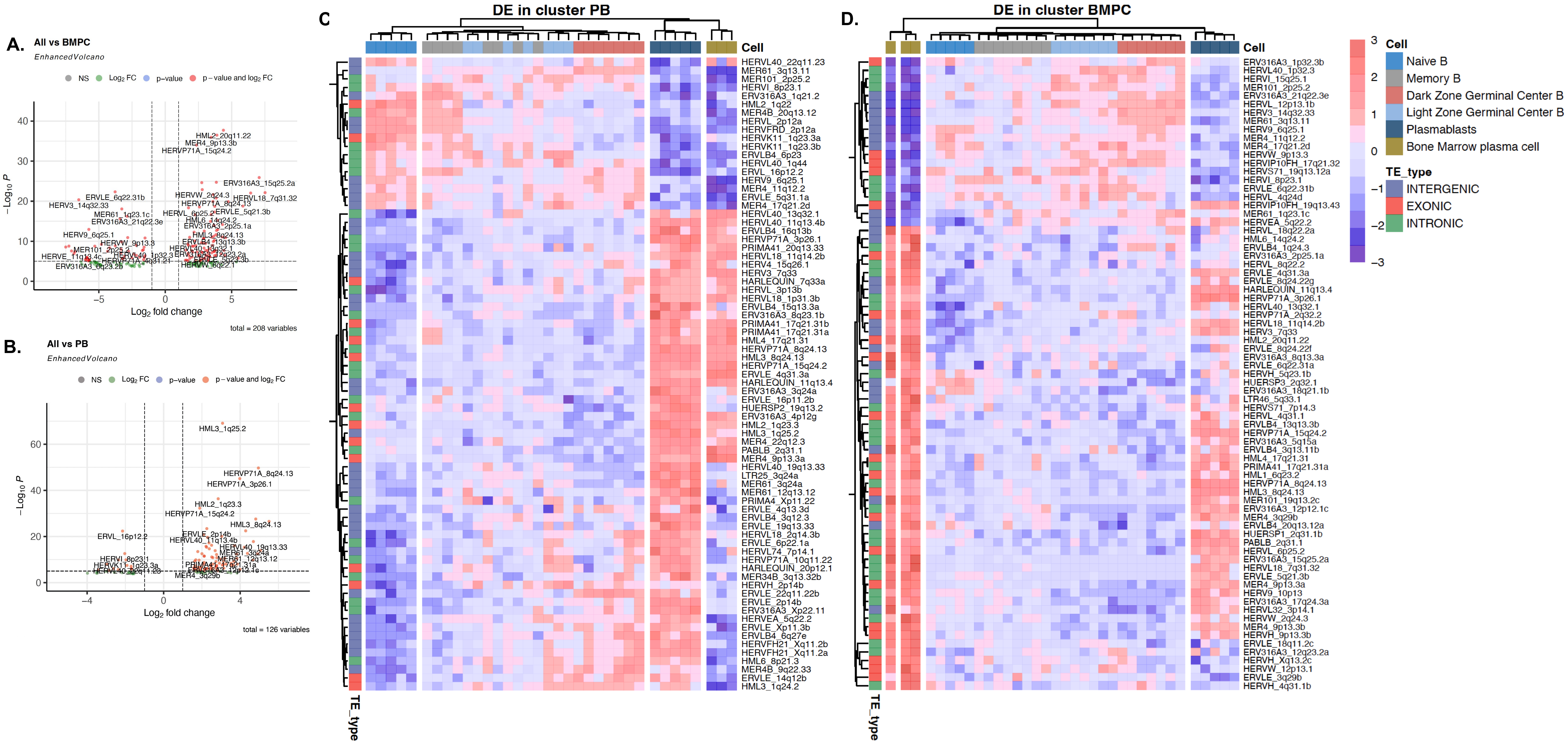

A.
All vs BMPC
EnhancedVolcano
NS
Log2 FC
p−value
p−value and log2 FC
HML2_20q11.22
MER4_9p13.3b
HERVP71A_15q24.2
ERV316A3_15q25.2a
ERVLE_6q22.31b
HERV3_14q32.33
HERVW_2q24.3
HERVP71A_8q24.13
HERVL18_7q31.32
MER61_1q23.1c
HERVL_6p25.2
ERVLE_5q21.3b
ERV316A3_21q22.3e
HML6_14q24.2
ERV316A3_2p25.1a
HERV9_6q25.1
HML3_8q24.13
HERVW_9p13.3
ERVLB4_13q13.3b
MER101_2p25.2
HERVL40_13q32.1
HERVE_11q13.4c
HERVL40_1p32.3
HERVP71A_4q31.21
ERV316A3_6q23.2b
HERVW_6q22.1
−Log10 P
Log2 fold change
total = 208 variables
B.
All vs PB
EnhancedVolcano
NS
Log2 FC
p−value
p−value and log2 FC
HML3_1q25.2
HERVP71A_8q24.13
HERVP71A_3p26.1
HML2_1q23.3
HERVP71A_15q24.2
ERVLE_2p14b
HML3_8q24.13
ERVL_16p12.2
HERVL40_11q13.4b
HERVL40_19q13.33
MER61_3q24a
HERVI_8p23.1
MER61_12q13.12
HERVK11_1q23.3a
PRIMA41_17q21.31a
HERVL40_22q11.23
ERV316A3_12p12.1c
MER4_3q29b
−Log10 P
Log2 fold change
total = 126 variables
C
DE in cluster PB
Cell
HERVL40_22q11.23
MER61_3q13.11
MER101_2p25.2
HERVI_8p23.1
ERV316A3_1q21.2
HML2_1q22
MER4B_20q13.12
HERVL_2p12a
HERVFRD_2p12a
HERVK11_1q23.3a
HERVK11_1q23.3b
ERVLB4_6p23
HERVL40_1q44
ERVL_16p12.2
HERV9_6q25.1
MER4_11q12.2
ERVLE_5q31.1a
MER4_17q21.2d
HERVL40_13q32.1
HERVL40_11q13.4b
ERVLB4_16q13b
HERVP71A_3p26.1
PRIMA41_20q13.33
HERVL18_11q14.2b
HERV4_15q26.1
HERV3_7q33
HARLEQUIN_7q33a
HERVL_3p13b
HERVL18_1p31.3b
ERVLB4_15q13.3a
ERV316A3_8q23.1b
PRIMA41_17q21.31b
PRIMA41_17q21.31a
HML4_17q21.31
HERVP71A_8q24.13
HML3_8q24.13
HERVP71A_15q24.2
ERVLE_4q31.3a
HARLEQUIN_11q13.4
ERV316A3_3q24a
ERVLE_16p11.2b
HUERSP2_19q13.2
ERV316A3_4p12g
HML2_1q23.3
HML3_1q25.2
MER4_22q12.3
PABLB_2q31.1
MER4_9p13.3a
HERVL40_19q13.33
LTR25_3q24a
MER61_3q24a
MER61_12q13.12
PRIMA4_Xp11.22
ERVLE_4q13.3d
ERVLB4_3q12.3
ERVLE_19q13.33
HERVL18_2q14.3b
ERVLE_6p22.1a
HERVL74_7p14.1
HERVP71A_10q11.22
HARLEQUIN_20p12.1
MER34B_3q13.32b
HERVH_2p14b
ERVLE_22q11.22b
ERVLE_2p14b
ERV316A3_Xp22.11
HERVEA_5q22.2
ERVLE_Xp11.3b
ERVLB4_6q27e
HERVFH21_Xq11.2b
HERVFH21_Xq11.2a
HML6_8p21.3
MER4B_9q22.33
ERVLE_14q12b
HML3_1q24.2
TE_type
D.
DE in cluster BMPC
Cell
ERV316A3_1p32.3b
HERVL40_1p32.3
HERVI_15q25.1
MER101_2p25.2
ERV316A3_21q22.3e
HERVL_12p13.1b
HERV3_14q32.33
MER61_3q13.11
HERV9_6q25.1
MER4_11q12.2
MER4_17q21.2d
HERVW_9p13.3
HERVIP10FH_17q21.32
HERVS71_19q13.12a
HERVI_8p23.1
ERVLE_6q22.31b
HERVL_4q24d
HERVIP10FH_19q13.43
MER61_1q23.1c
HERVEA_5q22.2
HERVL_18q22.2a
HML6_14q24.2
ERVLB4_1q24.3
ERV316A3_2p25.1a
HERVL_8q22.2
ERVLE_4q31.3a
ERVLE_8q24.22g
HARLEQUIN_11q13.4
HERVP71A_3p26.1
HERVL40_13q32.1
HERVP71A_2q32.2
HERVL18_11q14.2b
HERV3_7q33
HML2_20q11.22
ERVLE_8q24.22f
ERV316A3_8q13.3a
ERVLE_6q22.31a
HERVH_5q23.1b
HUERSP3_2q32.1
ERV316A3_18q21.1b
LTR46_5q33.1
HERVS71_7p14.3
HERVL_4q31.1
ERVLB4_13q13.3b
HERVP71A_15q24.2
ERV316A3_5q15a
ERVLB4_3q13.11b
HML4_17q21.31
PRIMA41_17q21.31a
HML1_6q23.2
HERVP71A_8q24.13
HML3_8q24.13
MER101_19q13.2c
ERV316A3_12p12.1c
MER4_3q29b
ERVLB4_20q13.12a
HUERSP1_2q31.1b
PABLB_2q31.1
HERVL_6p25.2
ERV316A3_15q25.2a
HERVL18_7q31.32
ERVLE_5q21.3b
MER4_9p13.3a
HERV9_10p13
ERV316A3_17q24.3a
HERVL32_3p14.1
HERVW_2q24.3
MER4_9p13.3b
HERVH_9p13.3b
ERVLE_18q11.2c
ERV316A3_12q23.2a
HERVH_Xq13.2c
HERVW_12p13.1
ERVLE_3q29b
HERVH_4q31.1b
TE_type
Cell
Naive B
Memory B
Dark Zone Germinal Center B
Light Zone Germinal Center B
Plasmablasts
Bone Marrow plasma cell
TE_type
INTERGENIC
EXONIC
INTRONIC

Supp Fig. 4

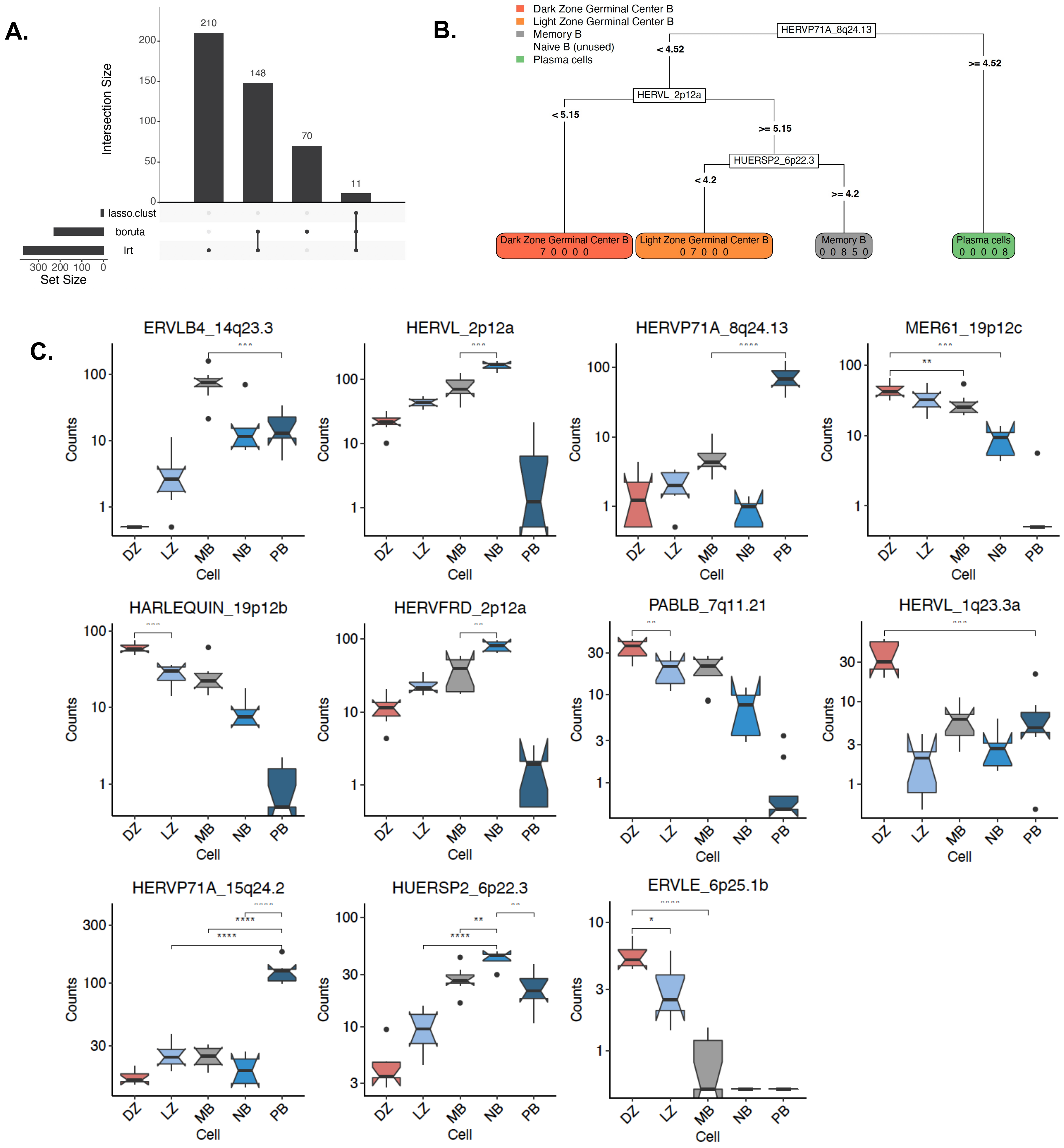
A.
Intersection Size
210
148
70
11
lasso.clust
boruta
lrt
Set Size
B.
Dark Zone Germinal Center B
Light Zone Germinal Center B
Memory B
Naive B (unused)
Plasma cells
HERVP71A_8q24.13
HERVL_2p12a
HUERSP2_6p22.3
C.
ERVLB4_14q23.3
HERVL_2p12a
HERVP71A_8q24.13
MER61_19p12c
HARLEQUIN_19p12b
HERVFRD_2p12a
PABLB_7q11.21
HERVL_1q23.3a
HERVP71A_15q24.2
HUERSP2_6p22.3
ERVLE_6p25.1b
Counts
Cell
DZ
LZ
MB
NB
PB

Supp Fig. 5

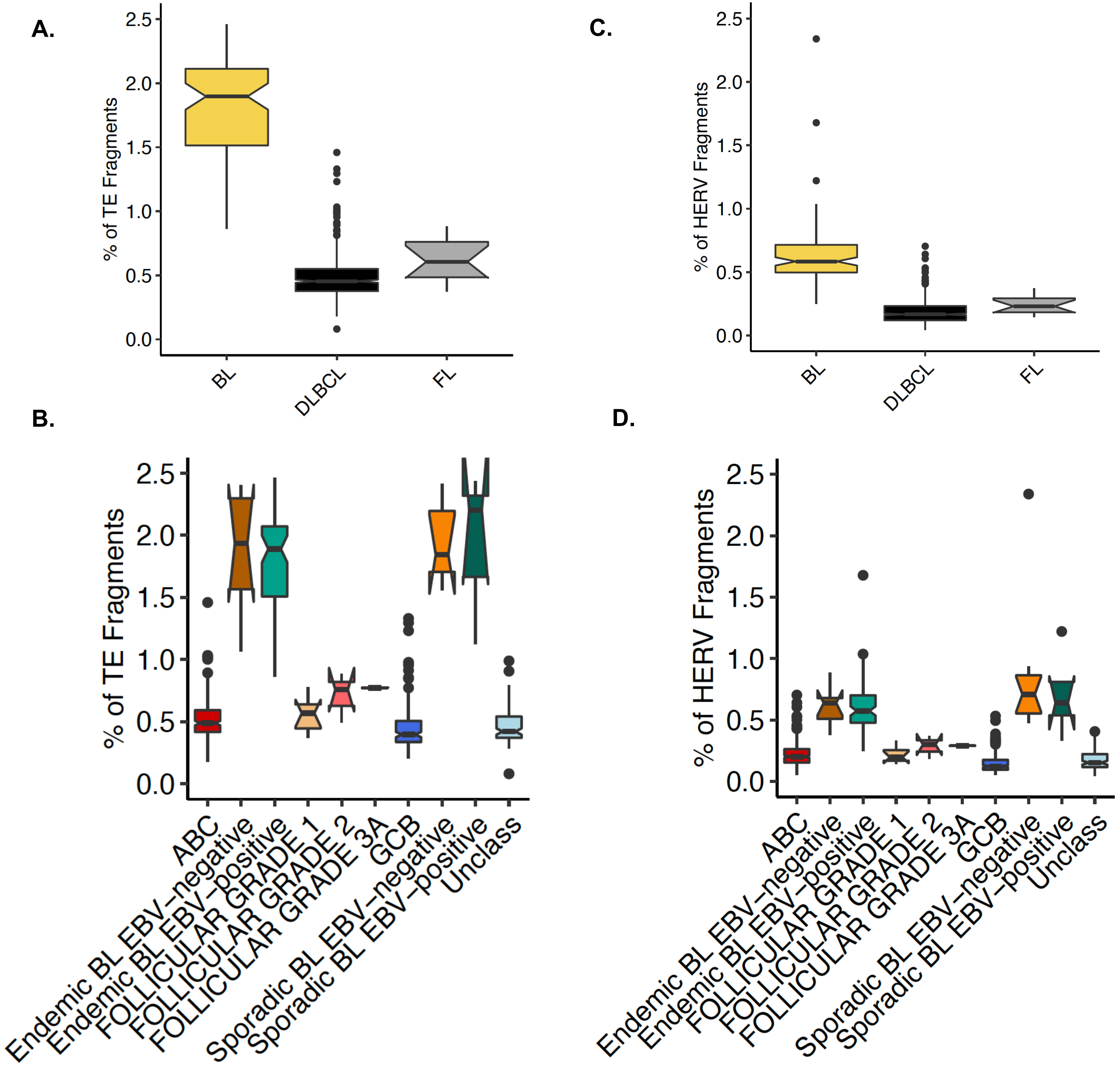

A.
% of TE Fragments
2.5
2.0
1.5
1.0
0.5
0.0
BL
DLBCL
FL
C.
% of HERV Fragments
2.5
2.0
1.5
1.0
0.5
0.0
BL
DLBCL
FL
B.
% of TE Fragments
2.5
2.0
1.5
1.0
0.5
0.0
ABC
Endemic BL EBV−negative
Endemic BL EBV−positive
FOLLICULAR GRADE 1
FOLLICULAR GRADE 2
FOLLICULAR GRADE 3A
GCB
Sporadic BL EBV−negative
Sporadic BL EBV−positive
Unclass
D.
% of HERV Fragments
2.5
2.0
1.5
1.0
0.5
0.0
ABC
Endemic BL EBV−negative
Endemic BL EBV−positive
FOLLICULAR GRADE 1
FOLLICULAR GRADE 2
FOLLICULAR GRADE 3A
GCB
Sporadic BL EBV−negative
Sporadic BL EBV−positive
Unclass

Supp Fig. 6

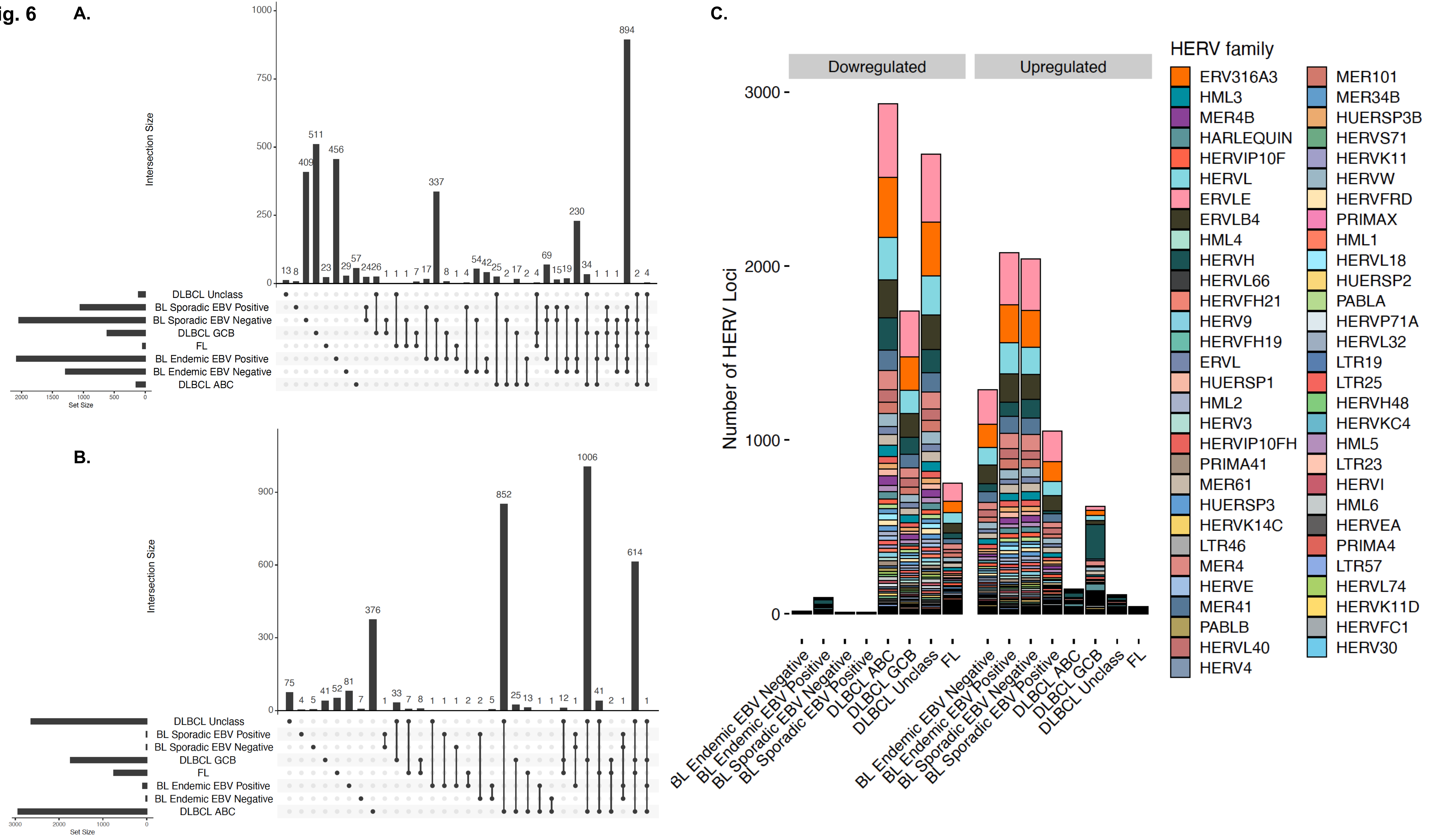
A.
Intersection Size
DLBCL Unclass
BL Sporadic EBV Positive
BL Sporadic EBV Negative
DLBCL GCB
FL
BL Endemic EBV Positive
BL Endemic EBV Negative
DLBCL ABC
Set Size
B.
Intersection Size
DLBCL Unclass
BL Sporadic EBV Positive
BL Sporadic EBV Negative
DLBCL GCB
FL
BL Endemic EBV Positive
BL Endemic EBV Negative
DLBCL ABC
Set Size
C.
Dowregulated
Upregulated
Number of HERV Loci
BL Endemic EBV Negative
BL Endemic EBV Positive
BL Sporadic EBV Negative
BL Sporadic EBV Positive
DLBCL ABC
DLBCL GCB
DLBCL Unclass
FL
HERV family
ERV316A3
HML3
MER4B
HARLEQUIN
HERVIP10F
HERVL
ERVLE
ERVLB4
HML4
HERVH
HERVL66
HERVFH21
HERV9
HERVFH19
ERVL
HUERSP1
HML2
HERV3
HERVIP10FH
PRIMA41
MER61
HUERSP3
HERVK14C
LTR46
MER4
HERVE
MER41
PABLB
HERVL40
HERV4
MER101
MER34B
HUERSP3B
HERVS71
HERVK11
HERVW
HERVFRD
PRIMAX
HML1
HERVL18
HUERSP2
PABLA
HERVP71A
HERVL32
LTR19
LTR25
HERVH48
HERVKC4
HML5
LTR23
HERVI
HML6
HERVEA
PRIMA4
LTR57
HERVL74
HERVK11D
HERVFC1
HERV30

Supp Fig. 7

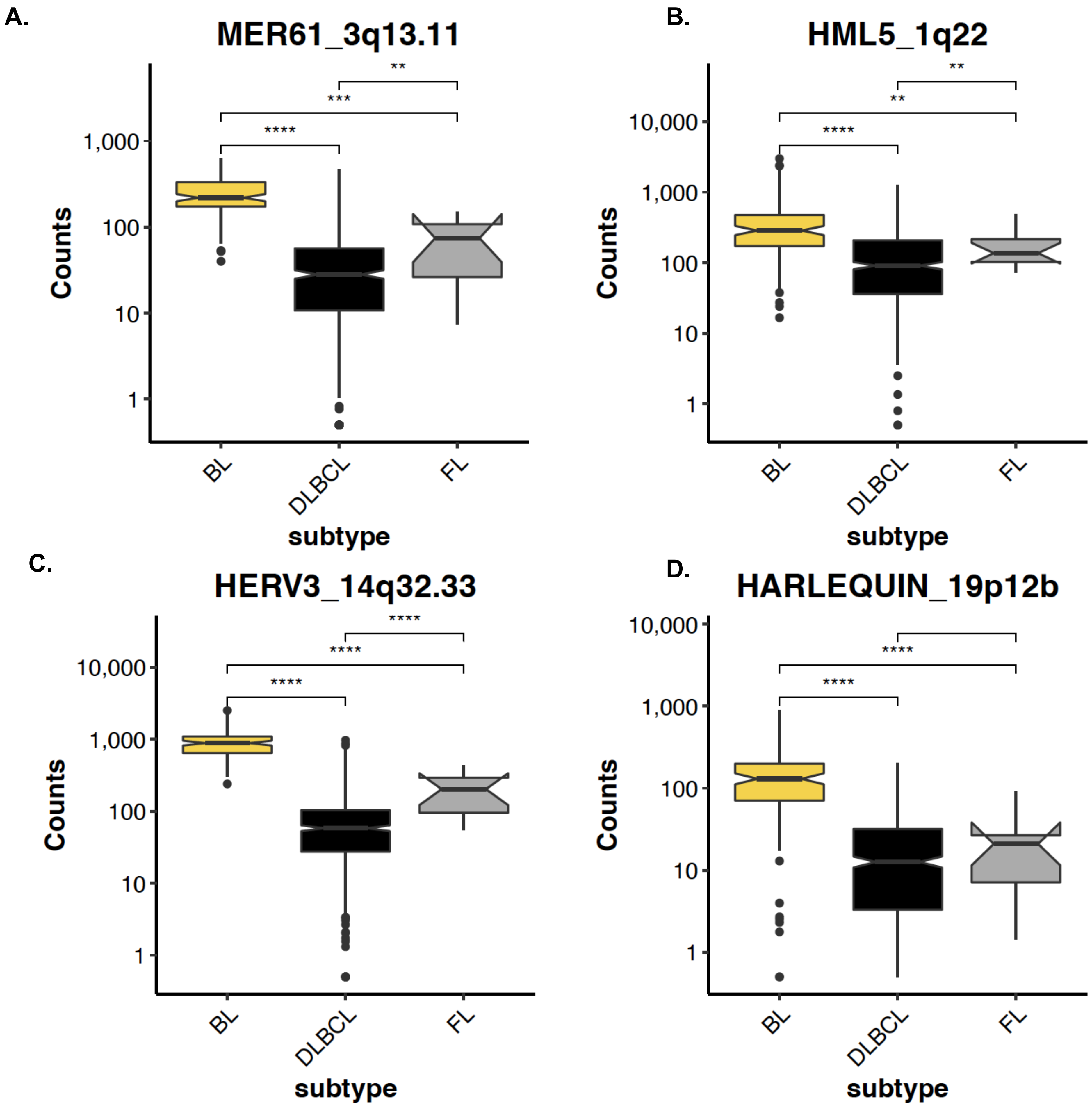
A.
MER61_3q13.11
Counts
1,000
100
10
1
****
***
**
BL
DLBCL
FL
subtype
B.
HML5_1q22
Counts
10,000
1,000
100
10
1
****
**
**
BL
DLBCL
FL
subtype
C.
HERV3_14q32.33
Counts
10,000
1,000
100
10
1
****
****
****
BL
DLBCL
FL
subtype
D.
HARLEQUIN_19p12b
Counts
10,000
1,000
100
10
1
****
****
BL
DLBCL
FL
subtype

Supp Fig. 8

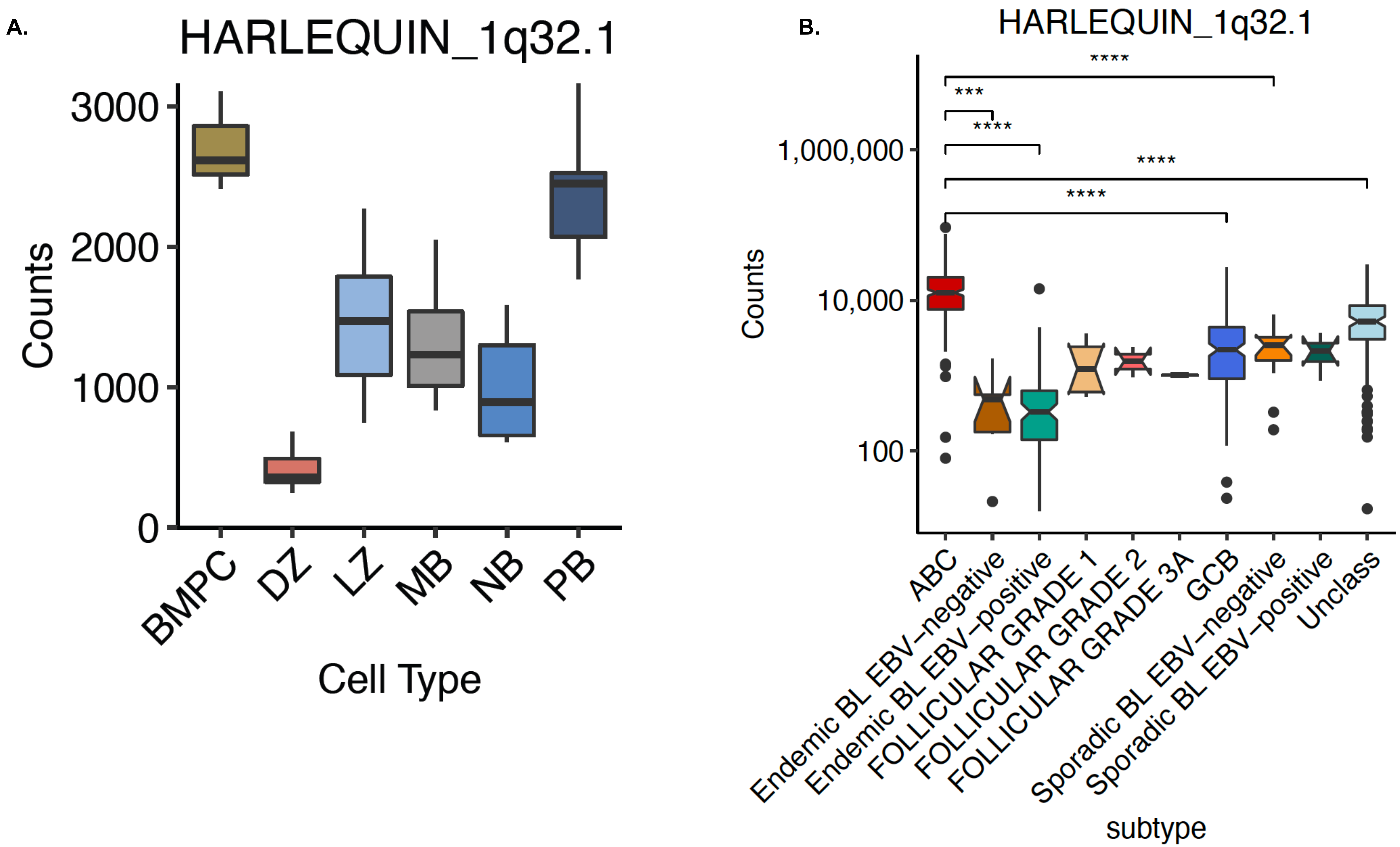
A.
HARLEQUIN_1q32.1
Counts
3000
2000
1000
0
BMPC
DZ
LZ
MB
NB
PB
Cell Type
B.
HARLEQUIN_1q32.1
****
***
****
****
****
1,000,000
10,000
100
Counts
ABC
Endemic BL EBV−negative
Endemic BL EBV−positive
FOLLICULAR GRADE 1
FOLLICULAR GRADE 2
FOLLICULAR GRADE 3A
GCB
Sporadic BL EBV−negative
Sporadic BL EBV−positive
Unclass
subtype

Supp Fig. 9

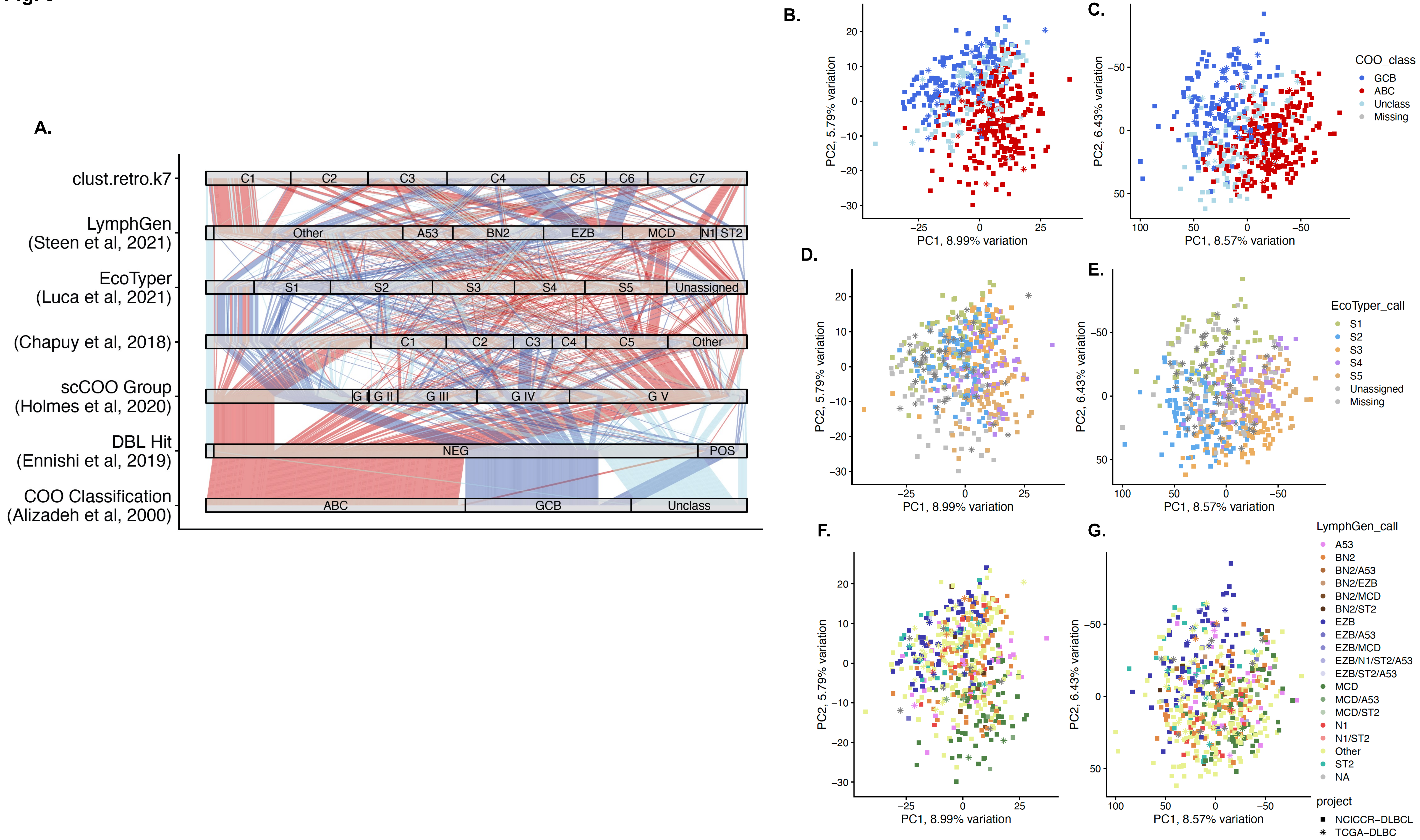

A.
clust.retro.k7
LymphGen (Steen et al, 2021)
EcoTyper (Luca et al, 2021)
(Chapuy et al, 2018)
scCOO Group (Holmes et al, 2020)
DBL Hit (Ennishi et al, 2019)
COO Classification (Alizadeh et al, 2000)
C1 C2 C3 C4 C5 C6 C7
Other A53 BN2 EZB MCD N1 ST2
S1 S2 S3 S4 S5 Unassigned
C1 C2 C3 C4 C5 Other
G I G II G III G IV G V
NEG POS
ABC GCB Unclass
B.
C.
D.
E.
F.
G.
PC1, 8.99% variation
PC2, 5.79% variation
PC1, 8.57% variation
PC2, 6.43% variation
COO_class
GCB
ABC
Unclass
Missing
EcoTyper_call
S1
S2
S3
S4
S5
Unassigned
Missing
LymphGen_call
A53
BN2
BN2/A53
BN2/EZB
BN2/MCD
BN2/ST2
EZB
EZB/A53
EZB/MCD
EZB/N1/ST2/A53
EZB/ST2/A53
MCD
MCD/A53
MCD/ST2
N1
N1/ST2
Other
ST2
NA
project
NCICCR–DLBCL
TCGA–DLBC

Supp Fig. 10

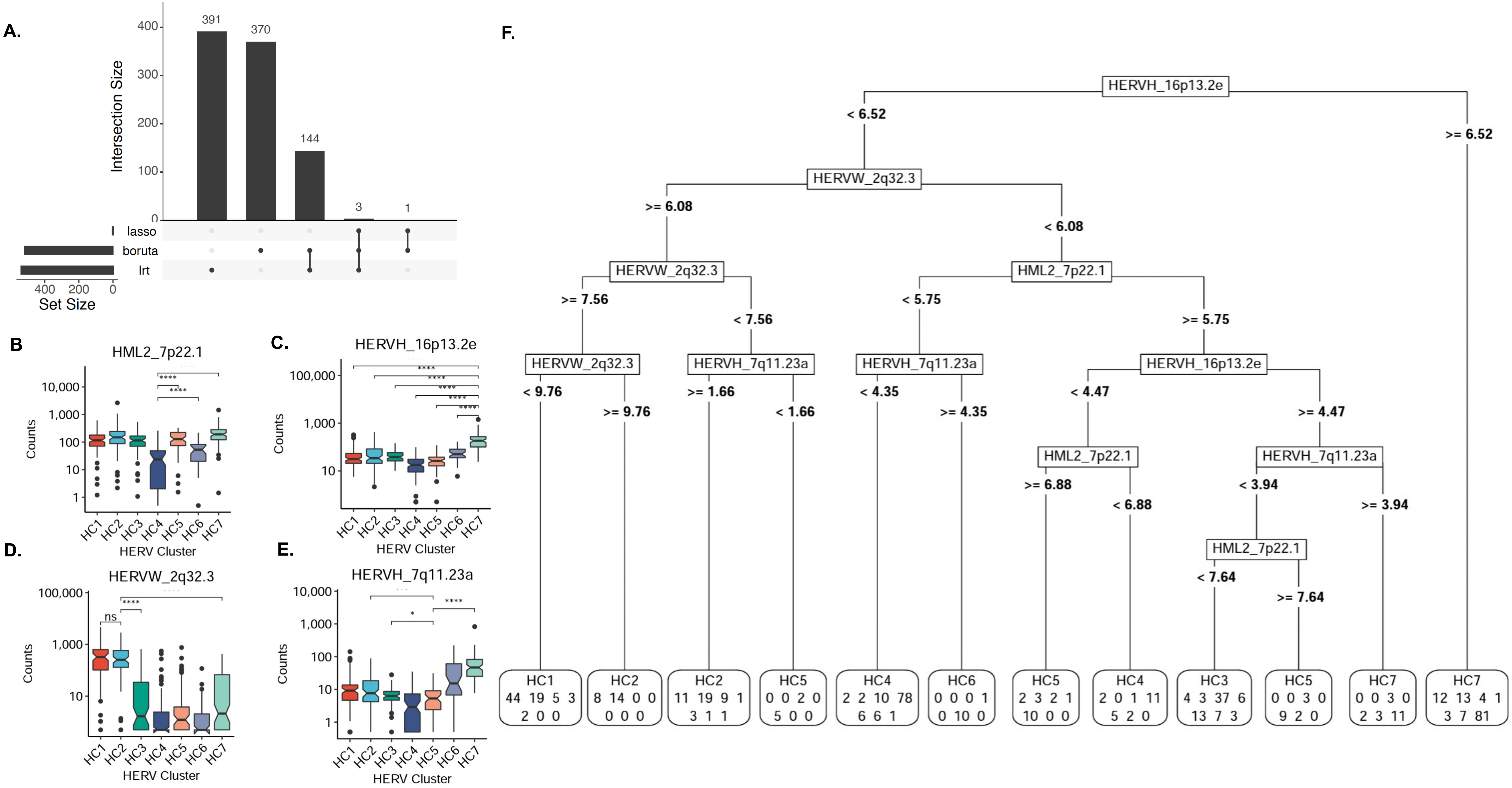
A.
Intersection Size
391
370
144
3
1
lasso
boruta
lrt
Set Size
B
HML2_7p22.1
Counts
HERV Cluster
C.
HERVH_16p13.2e
D.
HERVW_2q32.3
E.
HERVH_7q11.23a
F.
HERVH_16p13.2e
< 6.52
>= 6.52
HERVW_2q32.3
>= 6.08
< 6.08
HERVW_2q32.3
HML2_7p22.1
>= 7.56
< 7.56
< 5.75
>= 5.75
HERVW_2q32.3
HERVH_7q11.23a
HERVH_7q11.23a
HERVH_16p13.2e
< 9.76
>= 9.76
>= 1.66
< 1.66
< 4.35
>= 4.35
< 4.47
>= 4.47
HML2_7p22.1
HERVH_7q11.23a
>= 6.88
< 6.88
< 3.94
>= 3.94
HML2_7p22.1
< 7.64
>= 7.64
HC1 44 19 5 3 2 0 0
HC2 8 14 0 0 0 0 0
HC2 11 19 9 1 3 1 1
HC5 0 0 2 0 5 0 0
HC4 2 2 10 78 6 6 1
HC6 0 0 0 1 0 10 0
HC5 2 3 2 1 10 0 0
HC4 2 0 1 11 5 2 0
HC3 4 3 37 6 13 7 3
HC5 0 0 3 0 9 2 0
HC7 0 0 3 0 2 3 11
HC7 12 13 4 1 3 7 81

Supp Fig. 11

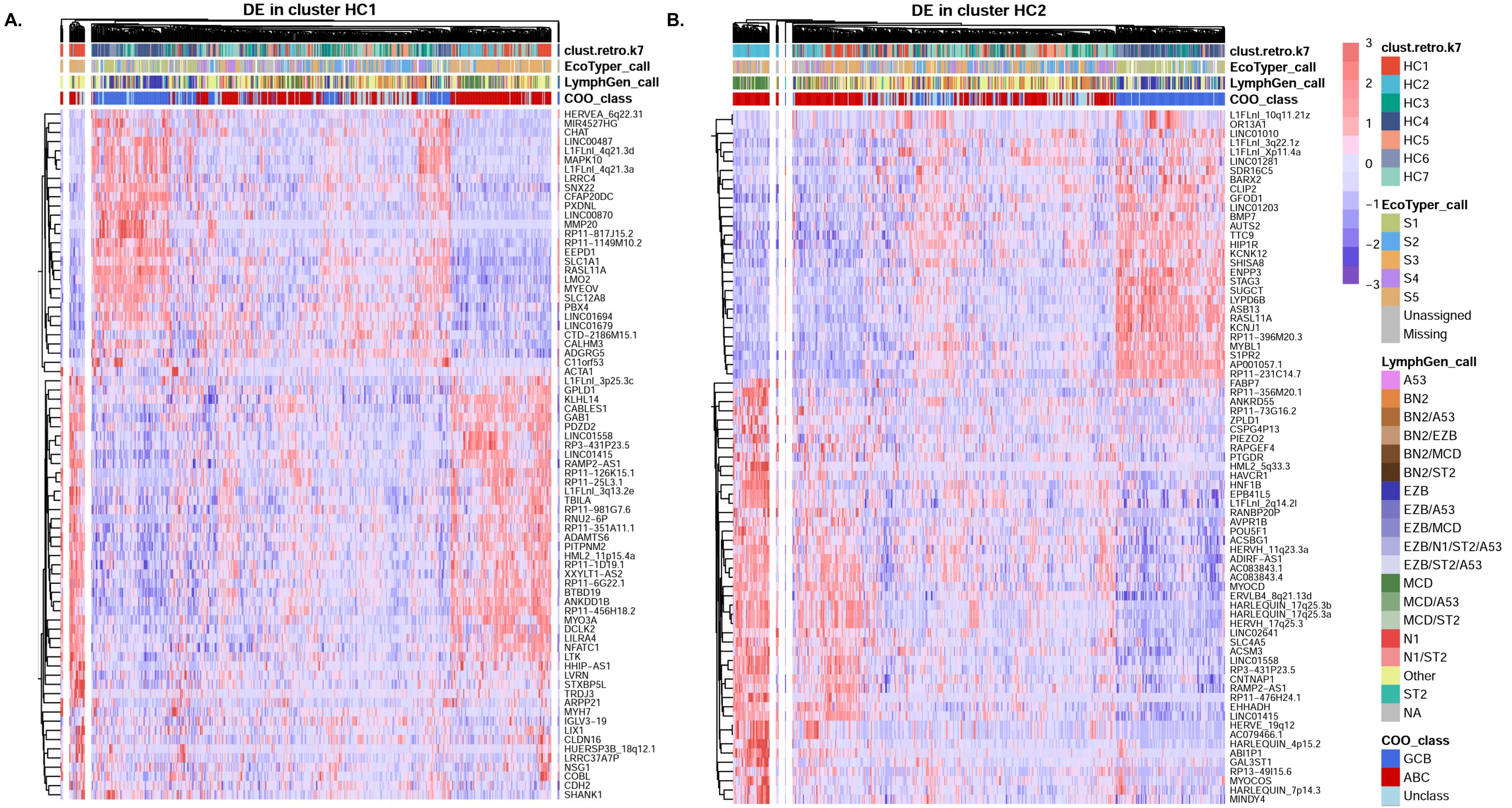

A.
DE in cluster HC1
clust.retro.k7
EcoTyper_call
LymphGen_call
COO_class
HERVEA_6q22.31
MIR4527HG
CHAT
LINC00487
L1FLnI_4q21.3d
MAPK10
L1FLnI_4q21.3a
LRRC4
SNX22
CFAP20DC
PXDNL
LINC00870
MMP20
RP11−817J15.2
RP11−1149M10.2
EEPD1
SLC1A1
RASL11A
LMO2
MYEOV
SLC12A8
PBX4
LINC01694
LINC01679
CTD−2186M15.1
CALHM3
ADGRG5
C11orf53
ACTA1
L1FLnI_3p25.3c
GPLD1
KLHL14
CABLES1
GAB1
PDZD2
LINC01558
RP3−431P23.5
LINC01415
RAMP2−AS1
RP11−126K15.1
RP11−25L3.1
L1FLnI_3q13.2e
TBILA
RP11−981G7.6
RNU2−6P
RP11−351A11.1
ADAMTS6
PITPNM2
HML2_11p15.4a
RP11−1D19.1
XXYLT1−AS2
RP11−6G22.1
BTBD19
ANKDD1B
RP11−456H18.2
MYO3A
DCLK2
LILRA4
NFATC1
LTK
HHIP−AS1
LVRN
STXBP5L
TRDJ3
ARPP21
MYH7
IGLV3−19
LIX1
CLDN16
HUERSP3B_18q12.1
LRRC37A7P
NSG1
COBL
CDH2
SHANK1
B.
DE in cluster HC2
clust.retro.k7
EcoTyper_call
LymphGen_call
COO_class
L1FLnI_10q11.21z
OR13A1
LINC01010
L1FLnI_3q22.1z
L1FLnI_Xp11.4a
LINC01281
SDR16C5
BARX2
CLIP2
GFOD1
LINC01203
BMP7
AUTS2
TTC9
HIP1R
KCNK12
SHISA8
ENPP3
STAG3
SUGCT
LYPD6B
ASB13
RASL11A
KCNJ1
RP11−396M20.3
MYBL1
S1PR2
AP001057.1
RP11−231C14.7
FABP7
RP11−356M20.1
ANKRD55
RP11−73G16.2
ZPLD1
CSPG4P13
PIEZO2
RAPGEF4
PTGDR
HML2_5q33.3
HAVCR1
HNF1B
EPB41L5
L1FLnI_2q14.2l
RANBP20P
AVPR1B
POU5F1
ACSBG1
HERVH_11q23.3a
ADIRF−AS1
AC083843.1
AC083843.4
MYOCD
ERVLB4_8q21.13d
HARLEQUIN_17q25.3b
HARLEQUIN_17q25.3a
HERVH_17q25.3
LINC02641
SLC4A5
ACSM3
LINC01558
RP3−431P23.5
CNTNAP1
RAMP2−AS1
RP11−476H24.1
EHHADH
LINC01415
HERVE_19q12
AC079466.1
HARLEQUIN_4p15.2
ABI1P1
GAL3ST1
RP13−49I15.6
MYOCOS
HARLEQUIN_7p14.3
MINDY4
3
2
1
0
−1
−2
−3
clust.retro.k7
HC1
HC2
HC3
HC4
HC5
HC6
HC7
EcoTyper_call
S1
S2
S3
S4
S5
Unassigned
Missing
LymphGen_call
A53
BN2
BN2/A53
BN2/EZB
BN2/MCD
BN2/ST2
EZB
EZB/A53
EZB/MCD
EZB/N1/ST2/A53
EZB/ST2/A53
MCD
MCD/A53
MCD/ST2
N1
N1/ST2
Other
ST2
NA
COO_class
GCB
ABC
Unclass

Supp Fig. 12

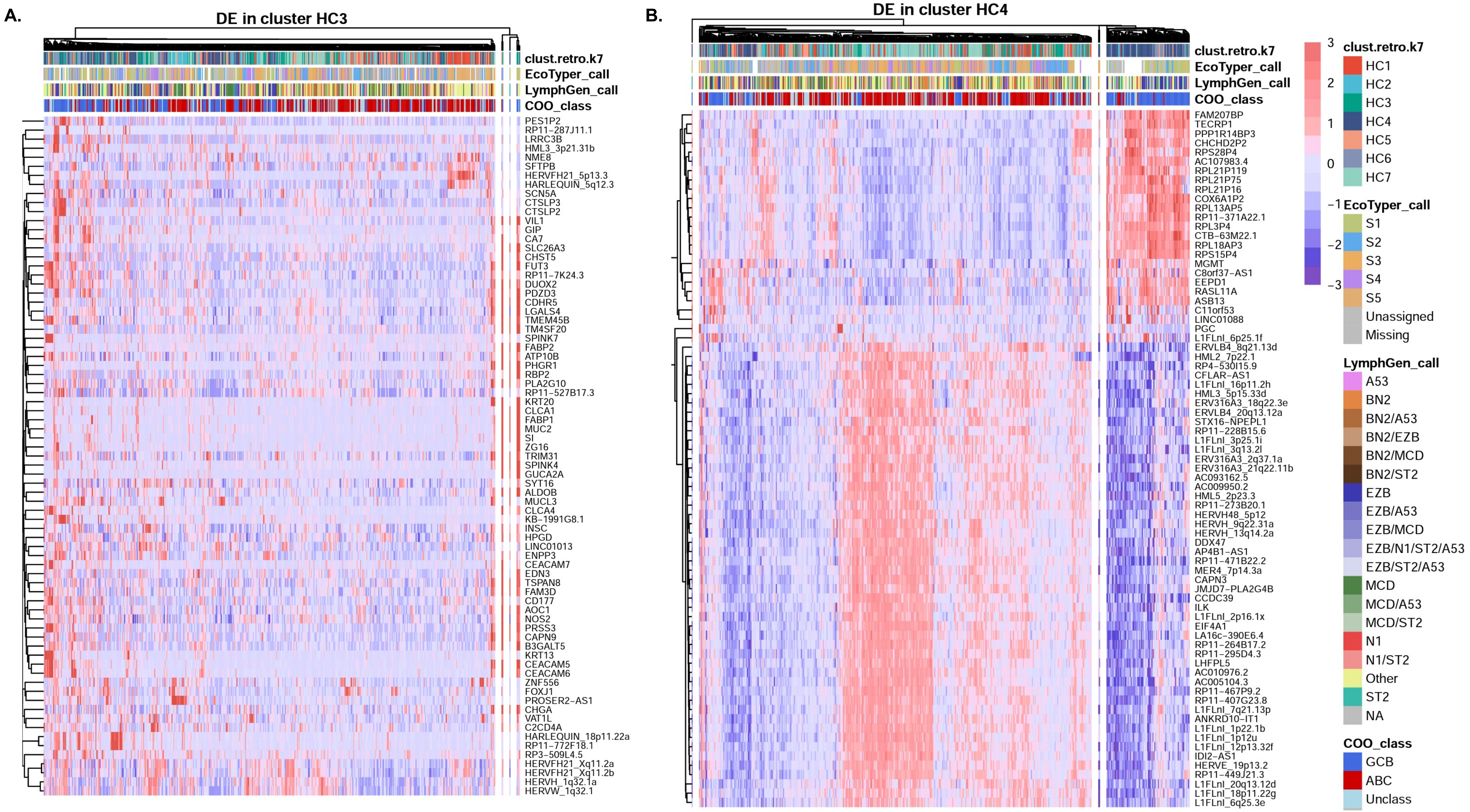
A.
DE in cluster HC3
clust.retro.k7
EcoTyper_call
LymphGen_call
COO_class
PES1P2
RP11−287J11.1
LRRC3B
HML3_3p21.31b
NME8
SFTPB
HERVFH21_5p13.3
HARLEQUIN_5q12.3
SCN5A
CTSLP3
CTSLP2
VIL1
GIP
CA7
SLC26A3
CHST5
FUT3
RP11−7K24.3
DUOX2
PDZD3
CDHR5
LGALS4
TMEM45B
TM4SF20
SPINK7
FABP2
ATP10B
PHGR1
RBP2
PLA2G10
RP11−527B17.3
KRT20
CLCA1
FABP1
MUC2
SI
ZG16
TRIM31
SPINK4
GUCA2A
SYT16
ALDOB
MUCL3
CLCA4
KB−1991G8.1
INSC
HPGD
LINC01013
ENPP3
CEACAM7
EDN3
TSPAN8
FAM3D
CD177
AOC1
NOS2
PRSS3
CAPN9
B3GALT5
KRT13
CEACAM5
CEACAM6
ZNF556
FOXJ1
PROSER2−AS1
CHGA
VAT1L
C2CD4A
HARLEQUIN_18p11.22a
RP11−772F18.1
RP3−509L4.5
HERVFH21_Xq11.2a
HERVFH21_Xq11.2b
HERVH_1q32.1a
HERVW_1q32.1
B.
DE in cluster HC4
clust.retro.k7
EcoTyper_call
LymphGen_call
COO_class
FAM207BP
TECRP1
PPP1R14BP3
CHCHD2P2
RPS28P4
AC107983.4
RPL21P119
RPL21P75
RPL21P16
COX6A1P2
RPL13AP5
RP11−371A22.1
RPL3P4
CTB−63M22.1
RPL18AP3
RPS15P4
MGMT
C8orf37−AS1
EEPD1
RASL11A
ASB13
C11orf53
LINC01088
PGC
L1FLnI_6p25.1f
ERVLB4_8q21.13d
HML2_7p22.1
RP4−530I15.9
CFLAR−AS1
L1FLnI_16p11.2h
HML3_5p15.33d
ERV316A3_18q22.3e
ERVLB4_20q13.12a
STX16−NPEPL1
RP11−228B15.6
L1FLnI_3p25.1i
L1FLnI_3q13.2l
ERV316A3_2q37.1a
ERV316A3_21q22.11b
AC093162.5
AC009950.2
HML5_2p23.3
RP11−273B20.1
HERVH48_5p12
HERVH_9q22.31a
HERVH_13q14.2a
DDX47
AP4B1−AS1
RP11−471B22.2
MER4_7p14.3a
CAPN3
JMJD7−PLA2G4B
CCDC39
ILK
L1FLnI_2p16.1x
EIF4A1
LA16c−390E6.4
RP11−264B17.2
RP11−295D4.3
LHFPL5
AC010976.2
AC005104.3
RP11−467P9.2
RP11−407G23.8
L1FLnI_7q21.13p
ANKRD10−IT1
L1FLnI_1p22.1b
L1FLnI_1p12u
L1FLnI_12p13.32f
IDI2−AS1
HERVE_19p13.2
RP11−449J21.3
L1FLnI_20q13.12d
L1FLnI_18p11.22g
L1FLnI_6q25.3e
3
2
1
0
−1
−2
−3
clust.retro.k7
HC1
HC2
HC3
HC4
HC5
HC6
HC7
EcoTyper_call
S1
S2
S3
S4
S5
Unassigned
Missing
LymphGen_call
A53
BN2
BN2/A53
BN2/EZB
BN2/MCD
BN2/ST2
EZB
EZB/A53
EZB/MCD
EZB/N1/ST2/A53
EZB/ST2/A53
MCD
MCD/A53
MCD/ST2
N1
N1/ST2
Other
ST2
NA
COO_class
GCB
ABC
Unclass

Supp Fig. 13

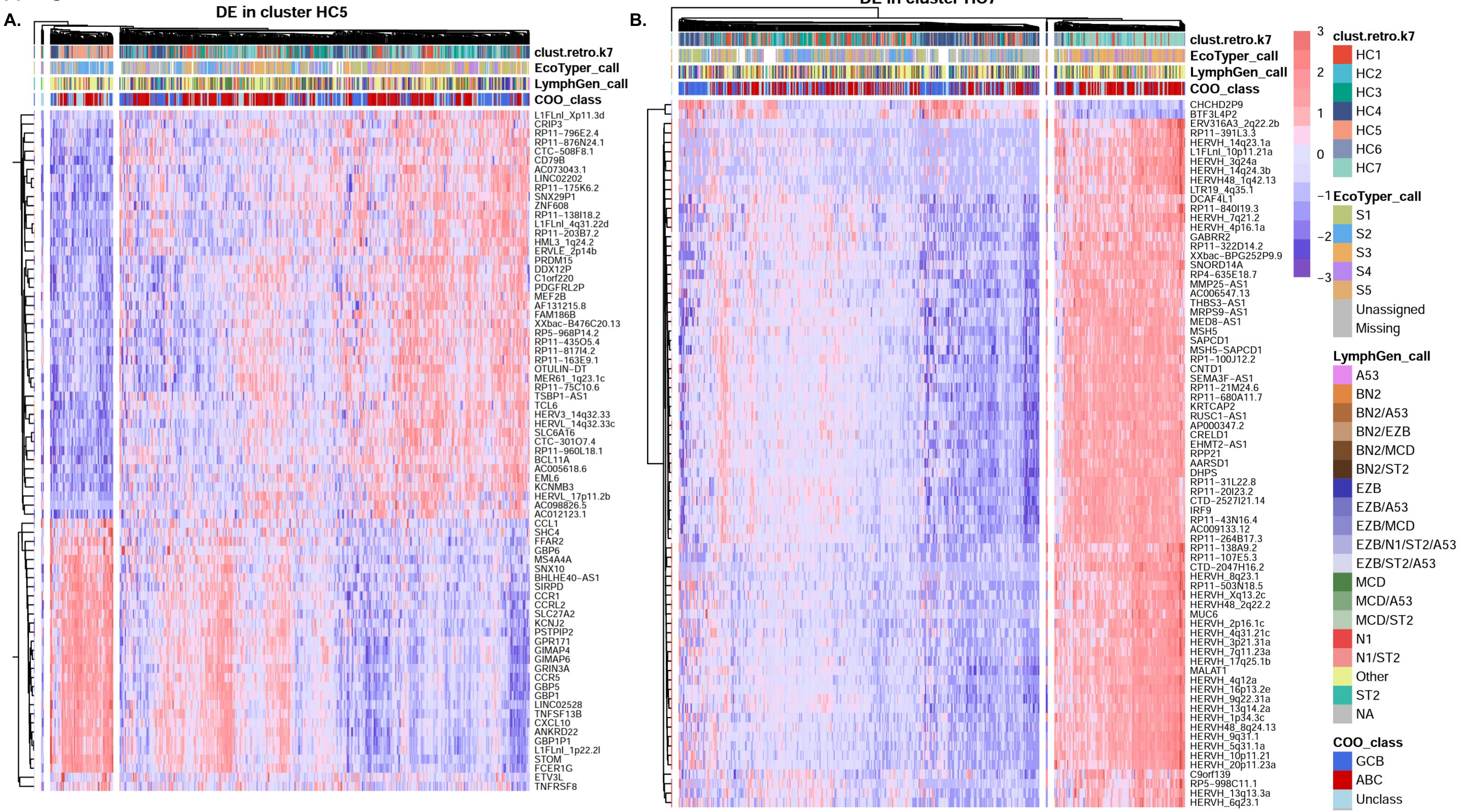
A.
DE in cluster HC5
clust.retro.k7
EcoTyper_call
LymphGen_call
COO_class
L1FLnI_Xp11.3d
CRIP3
RP11−796E2.4
RP11−876N24.1
CTC−508F8.1
CD79B
AC073043.1
LINC02202
RP11−175K6.2
SNX29P1
ZNF608
RP11−138I18.2
L1FLnI_4q31.22d
RP11−203B7.2
HML3_1q24.2
ERVLE_2p14b
PRDM15
DDX12P
C1orf220
PDGFRL2P
MEF2B
AF131215.8
FAM186B
XXbac−B476C20.13
RP5−968P14.2
RP11−435O5.4
RP11−817I4.2
RP11−163E9.1
OTULIN−DT
MER61_1q23.1c
RP11−75C10.6
TSBP1−AS1
TCL6
HERV3_14q32.33
HERVL_14q32.33c
SLC6A16
CTC−301O7.4
RP11−960L18.1
BCL11A
AC005618.6
EML6
KCNMB3
HERVL_17p11.2b
AC098826.5
AC012123.1
CCL1
SHC4
FFAR2
GBP6
MS4A4A
SNX10
BHLHE40−AS1
SIRPD
CCR1
CCRL2
SLC27A2
KCNJ2
PSTPIP2
GPR171
GIMAP4
GIMAP6
GRIN3A
CCR5
GBP5
GBP1
LINC02528
TNFSF13B
CXCL10
ANKRD22
GBP1P1
L1FLnI_1p22.2l
STOM
FCER1G
ETV3L
TNFRSF8
B.
DE in cluster HC7
clust.retro.k7
EcoTyper_call
LymphGen_call
COO_class
CHCHD2P9
BTF3L4P2
ERV316A3_2q22.2b
RP11−391L3.3
HERVH_14q23.1a
L1FLnI_10p11.21a
HERVH_3q24a
HERVH_14q24.3b
HERVH48_1q42.13
LTR19_4q35.1
DCAF4L1
RP11−840I19.3
HERVH_7q21.2
HERVH_4p16.1a
GABRR2
RP11−322D14.2
XXbac−BPG252P9.9
SNORD14A
RP4−635E18.7
MMP25−AS1
AC006547.13
THBS3−AS1
MRPS9−AS1
MED8−AS1
MSH5
SAPCD1
MSH5−SAPCD1
RP1−100J12.2
CNTD1
SEMA3F−AS1
RP11−21M24.6
RP11−680A11.7
KRTCAP2
RUSC1−AS1
AP000347.2
CRELD1
EHMT2−AS1
RPP21
AARSD1
DHPS
RP11−31L22.8
RP11−20I23.2
CTD−2527I21.14
IRF9
RP11−43N16.4
AC009133.12
RP11−264B17.3
RP11−138A9.2
RP11−107E5.3
CTD−2047H16.2
HERVH_8q23.1
RP11−503N18.5
HERVH_Xq13.2c
HERVH48_2q22.2
MUC6
HERVH_2p16.1c
HERVH_4q31.21c
HERVH_3p21.31a
HERVH_7q11.23a
HERVH_17q25.1b
MALAT1
HERVH_4q12a
HERVH_16p13.2e
HERVH_9q22.31a
HERVH_13q14.2a
HERVH_1p34.3c
HERVH48_8q24.13
HERVH_9q31.1
HERVH_5q31.1a
HERVH_10p11.21
HERVH_20p11.23a
C9orf139
RP5−998C11.1
HERVH_13q13.3a
HERVH_6q23.1
3
2
1
0
−1
−2
−3
clust.retro.k7
HC1
HC2
HC3
HC4
HC5
HC6
HC7
EcoTyper_call
S1
S2
S3
S4
S5
Unassigned
Missing
LymphGen_call
A53
BN2
BN2/A53
BN2/EZB
BN2/MCD
BN2/ST2
EZB
EZB/A53
EZB/MCD
EZB/N1/ST2/A53
EZB/ST2/A53
MCD
MCD/A53
MCD/ST2
N1
N1/ST2
Other
ST2
NA
COO_class
GCB
ABC
Unclass

Supp Fig. 14

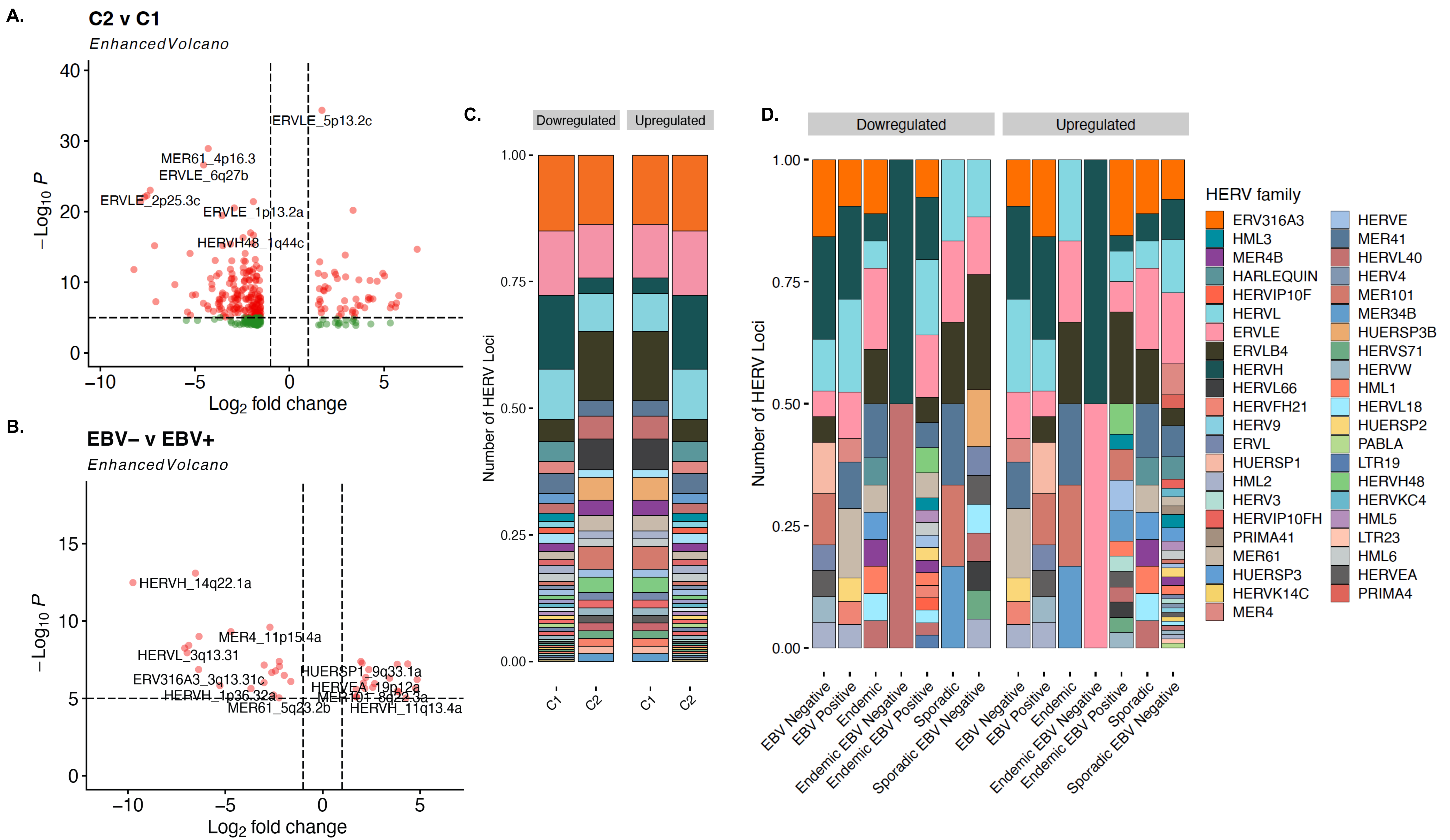
A.
C2 v C1
EnhancedVolcano
ERVLE_5p13.2c
MER61_4p16.3
ERVLE_6q27b
ERVLE_2p25.3c
ERVLE_1p13.2a
HERVH48_1q44c
−Log10 P
Log2 fold change
B.
EBV− v EBV+
EnhancedVolcano
HERVH_14q22.1a
MER4_11p15.4a
HERVL_3q13.31
ERV316A3_3q13.31c
HERVH_1p36.32a
MER61_5q23.2b
HUERSP1_9q33.1a
HERVEA_19p12a
MER101_8q22.3a
HERVH_11q13.4a
C.
Dowregulated
Upregulated
Number of HERV Loci
C1
C2
D.
Dowregulated
Upregulated
Number of HERV Loci
EBV Negative
EBV Positive
Endemic
Endemic EBV Negative
Endemic EBV Positive
Sporadic
Sporadic EBV Negative
HERV family
ERV316A3
HML3
MER4B
HARLEQUIN
HERVIP10F
HERVL
ERVLE
ERVLB4
HERVH
HERVL66
HERVFH21
HERV9
ERVL
HUERSP1
HML2
HERV3
HERVIP10FH
PRIMA41
MER61
HUERSP3
HERVK14C
MER4
HERVE
MER41
HERVL40
HERV4
MER101
MER34B
HUERSP3B
HERVS71
HERVW
HML1
HERVL18
HUERSP2
PABLA
LTR19
HERVH48
HERVKC4
HML5
LTR23
HML6
HERVEA
PRIMA4

Supp Fig. 15

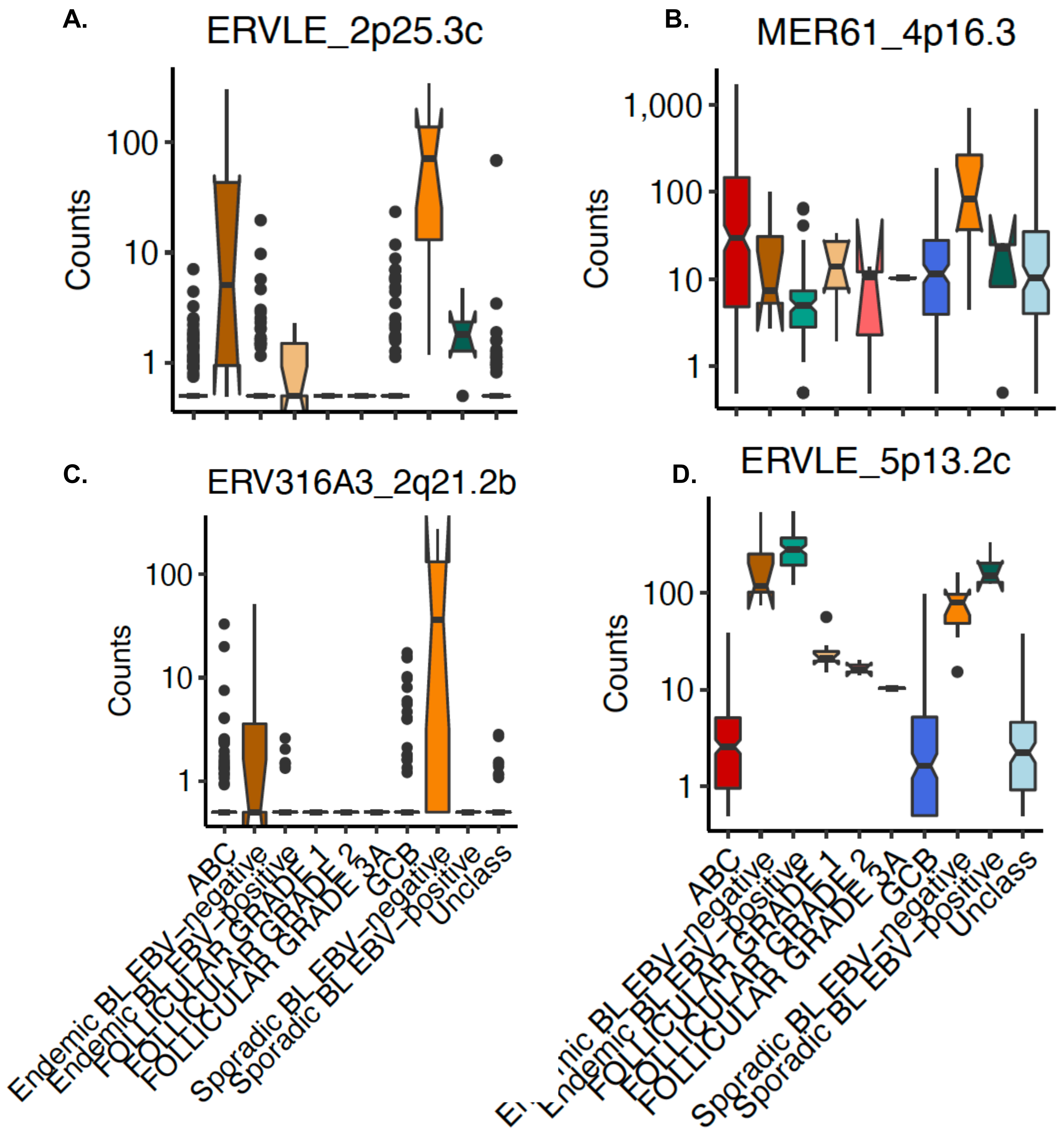
A.
ERVLE_2p25.3c
B.
MER61_4p16.3
C.
ERV316A3_2q21.2b
D.
ERVLE_5p13.2c
Counts
1
10
100
1,000
ABC
Endemic BL EBV–negative
Endemic BL EBV–positive
FOLLICULAR GRADE 1
FOLLICULAR GRADE 2
FOLLICULAR GRADE 3A
GCB
Sporadic BL EBV–negative
Sporadic BL EBV–positive
Unclass

Supp Fig. 16

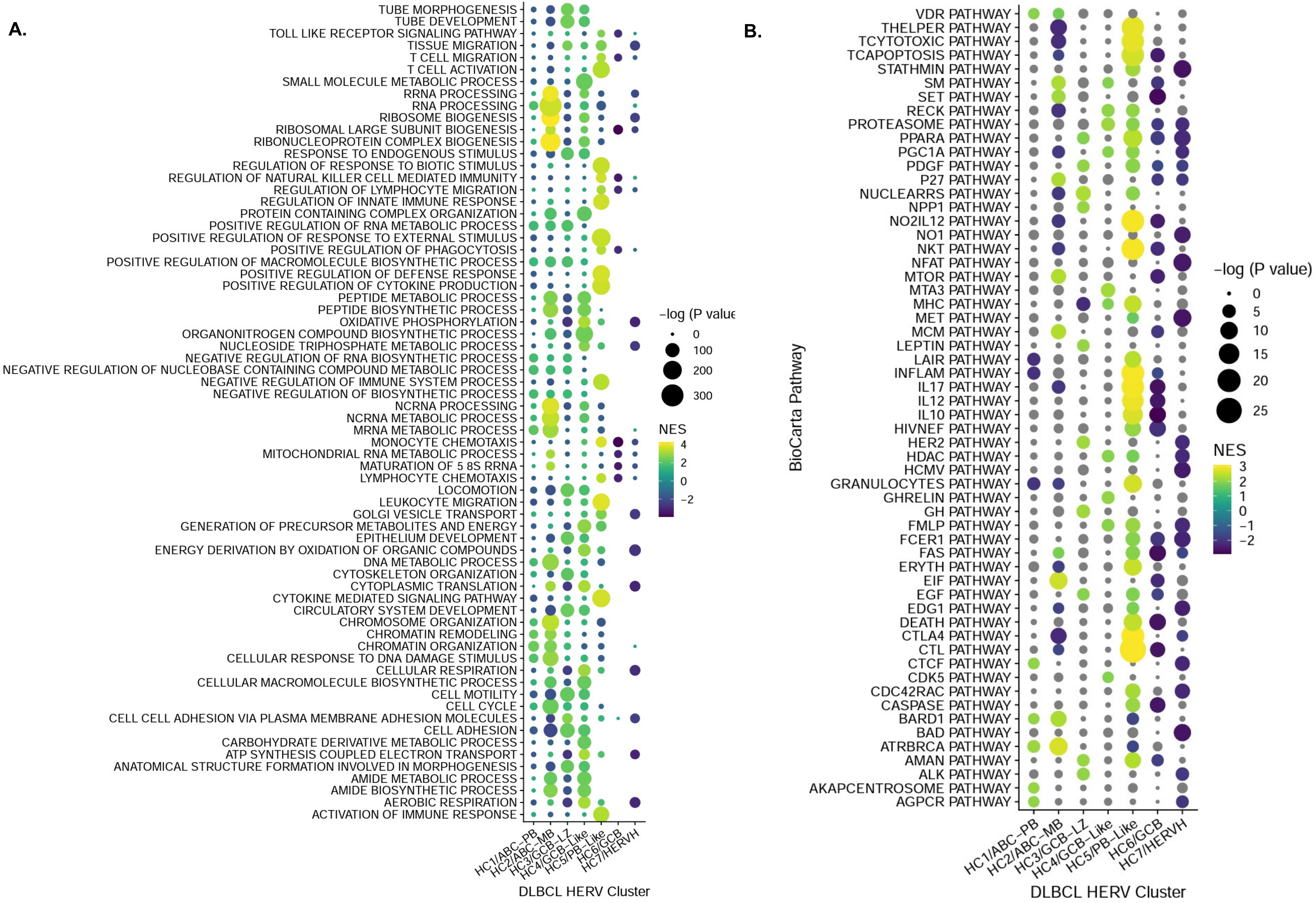
A.
TUBE MORPHOGENESIS
TUBE DEVELOPMENT
TOLL LIKE RECEPTOR SIGNALING PATHWAY
TISSUE MIGRATION
T CELL MIGRATION
T CELL ACTIVATION
SMALL MOLECULE METABOLIC PROCESS
RRNA PROCESSING
RNA PROCESSING
RIBOSOME BIOGENESIS
RIBOSOMAL LARGE SUBUNIT BIOGENESIS
RIBONUCLEOPROTEIN COMPLEX BIOGENESIS
RESPONSE TO ENDOGENOUS STIMULUS
REGULATION OF RESPONSE TO BIOTIC STIMULUS
REGULATION OF NATURAL KILLER CELL MEDIATED IMMUNITY
REGULATION OF LYMPHOCYTE MIGRATION
REGULATION OF INNATE IMMUNE RESPONSE
PROTEIN CONTAINING COMPLEX ORGANIZATION
POSITIVE REGULATION OF RNA METABOLIC PROCESS
POSITIVE REGULATION OF RESPONSE TO EXTERNAL STIMULUS
POSITIVE REGULATION OF PHAGOCYTOSIS
POSITIVE REGULATION OF MACROMOLECULE BIOSYNTHETIC PROCESS
POSITIVE REGULATION OF DEFENSE RESPONSE
POSITIVE REGULATION OF CYTOKINE PRODUCTION
PEPTIDE METABOLIC PROCESS
PEPTIDE BIOSYNTHETIC PROCESS
OXIDATIVE PHOSPHORYLATION
ORGANONITROGEN COMPOUND BIOSYNTHETIC PROCESS
NUCLEOSIDE TRIPHOSPHATE METABOLIC PROCESS
NEGATIVE REGULATION OF RNA BIOSYNTHETIC PROCESS
NEGATIVE REGULATION OF NUCLEOBASE CONTAINING COMPOUND METABOLIC PROCESS
NEGATIVE REGULATION OF IMMUNE SYSTEM PROCESS
NEGATIVE REGULATION OF BIOSYNTHETIC PROCESS
NCRNA PROCESSING
NCRNA METABOLIC PROCESS
MRNA METABOLIC PROCESS
MONOCYTE CHEMOTAXIS
MITOCHONDRIAL RNA METABOLIC PROCESS
MATURATION OF 5 8S RRNA
LYMPHOCYTE CHEMOTAXIS
LOCOMOTION
LEUKOCYTE MIGRATION
GOLGI VESICLE TRANSPORT
GENERATION OF PRECURSOR METABOLITES AND ENERGY
EPITHELIUM DEVELOPMENT
ENERGY DERIVATION BY OXIDATION OF ORGANIC COMPOUNDS
DNA METABOLIC PROCESS
CYTOSKELETON ORGANIZATION
CYTOPLASMIC TRANSLATION
CYTOKINE MEDIATED SIGNALING PATHWAY
CIRCULATORY SYSTEM DEVELOPMENT
CHROMOSOME ORGANIZATION
CHROMATIN REMODELING
CHROMATIN ORGANIZATION
CELLULAR RESPONSE TO DNA DAMAGE STIMULUS
CELLULAR RESPIRATION
CELLULAR MACROMOLECULE BIOSYNTHETIC PROCESS
CELL MOTILITY
CELL CYCLE
CELL CELL ADHESION VIA PLASMA MEMBRANE ADHESION MOLECULES
CELL ADHESION
CARBOHYDRATE DERIVATIVE METABOLIC PROCESS
ATP SYNTHESIS COUPLED ELECTRON TRANSPORT
ANATOMICAL STRUCTURE FORMATION INVOLVED IN MORPHOGENESIS
AMIDE METABOLIC PROCESS
AMIDE BIOSYNTHETIC PROCESS
AEROBIC RESPIRATION
ACTIVATION OF IMMUNE RESPONSE
GO BP Pathway
HC1/ABC−PB
HC2/ABC−MB
HC3/GCB−LZ
HC4/GCB−Like
HC5/PB−Like
HC6/GCB
HC7/HERVH
DLBCL HERV Cluster
−log (P value
0
100
200
300
NES
4
2
0
−2
B.
VDR PATHWAY
THELPER PATHWAY
TCYTOTOXIC PATHWAY
TCAPOPTOSIS PATHWAY
STATHMIN PATHWAY
SM PATHWAY
SET PATHWAY
RECK PATHWAY
PROTEASOME PATHWAY
PPARA PATHWAY
PGC1A PATHWAY
PDGF PATHWAY
P27 PATHWAY
NUCLEARRS PATHWAY
NPP1 PATHWAY
NO2IL12 PATHWAY
NO1 PATHWAY
NKT PATHWAY
NFAT PATHWAY
MTOR PATHWAY
MTA3 PATHWAY
MHC PATHWAY
MET PATHWAY
MCM PATHWAY
LEPTIN PATHWAY
LAIR PATHWAY
INFLAM PATHWAY
IL17 PATHWAY
IL12 PATHWAY
IL10 PATHWAY
HIVNEF PATHWAY
HER2 PATHWAY
HDAC PATHWAY
HCMV PATHWAY
GRANULOCYTES PATHWAY
GHRELIN PATHWAY
GH PATHWAY
FMLP PATHWAY
FCER1 PATHWAY
FAS PATHWAY
ERYTH PATHWAY
EIF PATHWAY
EGF PATHWAY
EDG1 PATHWAY
DEATH PATHWAY
CTLA4 PATHWAY
CTL PATHWAY
CTCF PATHWAY
CDK5 PATHWAY
CDC42RAC PATHWAY
CASPASE PATHWAY
BARD1 PATHWAY
BAD PATHWAY
ATRBRCA PATHWAY
AMAN PATHWAY
ALK PATHWAY
AKAPCENTROSOME PATHWAY
AGPCR PATHWAY
BioCarta Pathway
HC1/ABC−PB
HC2/ABC−MB
HC3/GCB−LZ
HC4/GCB−Like
HC5/PB−Like
HC6/GCB
HC7/HERVH
DLBCL HERV Cluster
−log (P value)
0
5
10
15
20
25
NES
3
2
1
0
−1
−2

Supp Fig. 17

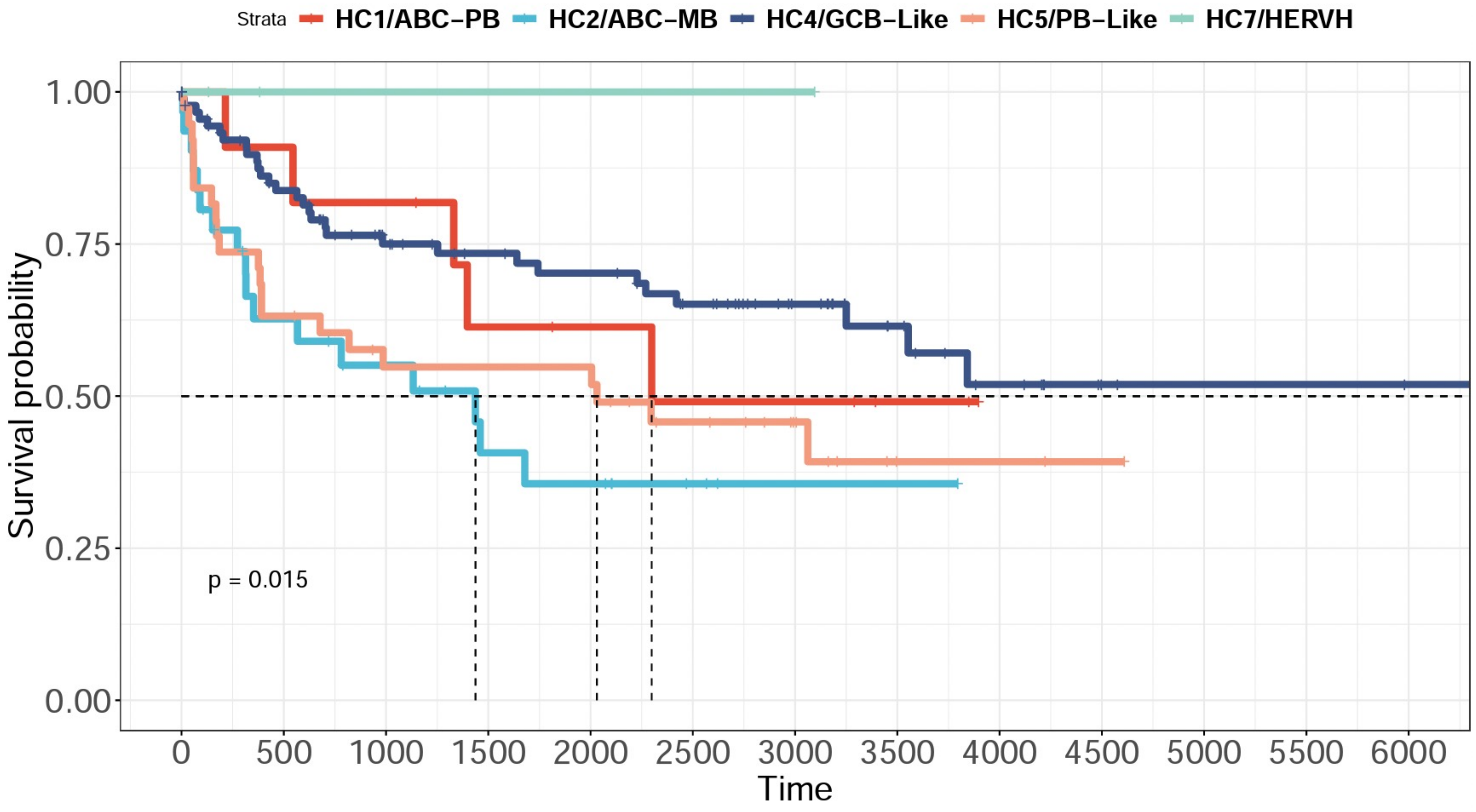

Strata
HC1/ABC−PB
HC2/ABC−MB
HC4/GCB−Like
HC5/PB−Like
HC7/HERVH
Survival probability
1.00
0.75
0.50
0.25
0.00
p = 0.015
0
500
1000
1500
2000
2500
3000
3500
4000
4500
5000
5500
6000
Time

Supplementary Tables

	Number of Genes	Number of LINEs	Number of HERVs
**DLBLC (n=529)**	36,246	8,886	4,567
**BL (n=113)**	34,453	16,350	4,099
**FL (n=12)**	27,908	2,593	2,068
**B-AG (n=35)**	25,629	1,520	1,118
**B-HM (n=17)**	23,709	1,939	1,464

Supplementary Table 1: Number of coding genes, LINEs, and HERV loci remaining in each dataset after filtering

Cell Type	baseMean	log2FoldChange	lfcSE	pvalue	padj	locus
BMPC	10.8887346	4.37784863	0.33767889	1.94E-38	2.17E-35	HML2_20q11.22
BMPC	46.2145428	3.88647483	0.30492399	3.29E-37	1.84E-34	MER4_9p13.3a
BMPC	20.9878326	3.44465987	0.27234532	1.15E-36	4.26E-34	MER4_9p13.3b
BMPC	44.8270989	2.35333027	0.19148483	1.03E-34	2.86E-32	HERVP71A_15q24.2
BMPC	6.79069424	7.07315797	0.66226202	1.26E-26	2.81E-24	ERV316A3_15q25.2a
BMPC	9.19435008	3.84511792	0.3688537	1.92E-25	3.56E-23	ERV316A3_5q15a
BMPC	55.7281107	2.74673438	0.26396941	2.34E-25	3.73E-23	ERVLE_4q31.3a
BMPC	36.4028759	2.78409297	0.27798618	1.31E-23	1.82E-21	HERVW_2q24.3
BMPC	166.558057	-3.778879	0.38213487	4.65E-23	5.76E-21	ERVLE_6q22.31b
BMPC	11.2035607	7.51598707	0.76360265	7.36E-23	8.21E-21	HERVL18_7q31.32
DZ	314.964823	2.94489674	0.2194209	4.54E-41	5.06E-38	HERV3_14q32.33
DZ	11.0315708	4.19079876	0.34262119	2.11E-34	1.18E-31	HERVFH21_1p36.31
DZ	69.0711909	3.22244667	0.29935487	5.06E-27	1.88E-24	MER61_3q13.11
DZ	36.1160058	3.24496308	0.30403643	1.36E-26	3.80E-24	HERVFH21_7q11.21
DZ	24.5969539	3.75589702	0.36076565	2.21E-25	4.93E-23	HARLEQUIN_19p12b
DZ	10.7288541	3.31204897	0.33535332	5.27E-23	9.80E-21	HERVL_1q23.3a
DZ	7.64190534	4.00058561	0.41853948	1.20E-21	1.90E-19	MER4_2q14.1b
DZ	99.9825746	3.81395034	0.3995716	1.36E-21	1.90E-19	HERVL_12p13.1b
DZ	6.50233386	4.54297017	0.47952142	2.69E-21	3.34E-19	ERV316A3_Xp21.2
DZ	260.455902	1.50529524	0.15925132	3.31E-21	3.69E-19	HML5_12q23.1
LZ	112.252844	1.75835201	0.16973012	3.78E-25	4.22E-22	HERVI_15q25.1
LZ	26.4997086	3.21164587	0.31634322	3.23E-24	1.80E-21	ERVLB4_6p23
LZ	51.5932719	2.17517652	0.2288356	1.99E-21	7.41E-19	ERVLB4_8p21.3b
LZ	74.7588178	2.24408657	0.24562613	6.47E-20	1.80E-17	MER101_2p25.2
LZ	198.648314	1.63212081	0.17962949	1.03E-19	1.91E-17	HERVH_2p14b
LZ	314.964823	1.99906159	0.21988242	9.77E-20	1.91E-17	HERV3_14q32.33
LZ	41.7091526	3.78914004	0.44762951	2.56E-17	2.86E-15	ERV316A3_2q35i
LZ	15.9321312	2.64051774	0.32523319	4.71E-16	4.37E-14	MER41_4q31.1
LZ	99.9825746	3.1939485	0.39996917	1.40E-15	1.20E-13	HERVL_12p13.1b
LZ	76.5627064	1.52153044	0.19093247	1.60E-15	1.27E-13	HML5_3q26.2

MB	25.5376126	4.01253318	0.44604159	2.34E-19	2.41E-16	ERVLB4_14q23.3
MB	54.6747308	2.32886096	0.31650359	1.87E-13	9.60E-11	HERVL_2p12a
MB	69.0711909	2.0987513	0.29658573	1.48E-12	4.28E-10	MER61_3q13.11
MB	19.8407038	2.43669552	0.34513764	1.66E-12	4.28E-10	HARLEQUIN_5q33.3
MB	4.11604419	3.50125529	0.50611128	4.58E-12	9.43E-10	ERVLE_9q21.31d
MB	23.2968619	2.45890083	0.36513474	1.65E-11	2.83E-09	MER61_19p12c
MB	26.8514584	2.19928295	0.32864405	2.20E-11	3.24E-09	HERVFRD_2p12a
MB	24.5969539	2.31764342	0.36077492	1.33E-10	1.52E-08	HARLEQUIN_19p12b
MB	68.1825026	1.69218373	0.2679894	2.71E-10	2.54E-08	HML2_1q22
MB	16.0936966	2.17570587	0.37409464	6.03E-09	4.77E-07	PABLB_7q11.21
NB	260.161745	-5.3430997	0.48496785	3.15E-28	3.51E-25	HERVL18_11q14.2b
NB	68.1825026	3.36527491	0.31318367	6.23E-27	3.47E-24	HML2_1q22
NB	54.6747308	3.62913079	0.34056663	1.63E-26	6.07E-24	HERVL_2p12a
NB	26.8514584	3.52390742	0.35350931	2.10E-23	5.84E-21	HERVFRD_2p12a
NB	198.648314	-2.1717859	0.22052682	6.98E-23	1.56E-20	HERVH_2p14b
NB	97.3158729	-1.8246397	0.20821681	1.90E-18	3.53E-16	HERVH_15q26.3b
NB	102.48819	-2.8722752	0.33270862	5.98E-18	9.52E-16	HERVP71A_3p26.1
NB	102.36481	-2.0725408	0.24705674	4.91E-17	6.84E-15	ERVLE_2p14b
NB	123.700933	2.16855566	0.2630104	1.65E-16	2.04E-14	HERV9_6q25.1
NB	47.5824101	1.77030829	0.21806237	4.73E-16	5.27E-14	HERVK11_1q23.3a
PB	160.78374	3.08148225	0.17442372	7.58E-70	8.45E-67	HML3_1q25.2
PB	18.6471599	4.95495287	0.33177381	1.96E-50	1.09E-47	HERVP71A_8q24.13
PB	102.48819	3.98135965	0.28015381	7.79E-46	2.90E-43	HERVP71A_3p26.1
PB	74.2730714	2.85271936	0.22421539	4.40E-37	1.23E-34	HML2_1q23.3
PB	44.8270989	1.88966056	0.15791043	5.31E-33	1.18E-30	HERVP71A_15q24.2
PB	18.9632769	4.81596924	0.43653806	2.67E-28	4.97E-26	HML3_8q24.13
PB	7.1632018	5.51179855	0.5095672	2.87E-27	4.57E-25	ERVLB4_15q13.3a
PB	102.36481	2.25871145	0.22286633	3.87E-24	5.39E-22	ERVLE_2p14b
PB	13.8075309	4.28984401	0.43258816	3.52E-23	4.36E-21	LTR25_3q24a
PB	194.639084	-2.1472848	0.21704713	4.46E-23	4.97E-21	ERVL_16p12.2

Supplementary Table 2: Top 10 differentially expressed HERVs in each B-cell subtype in the B-AG dataset

**DZ**	**LZ**	**PB**	**BMPC**	**NB**	**MB**
NOL9	TMEM131L	CALCOCO1	SPTBN2	CD72	SESN3
BCL7A	CD22	RP11-16E12.2	SCARB2	COL19A1	PARP15
DCK	SWAP70	FBH1	SPAG4	MACF1	L1FLnI_3q13.2a
TMEM131L	LCP1	SLC17A9	FOXO3	TREML2	FCRL4

DPY19L2P2	RFTN1	WDR45	ABCB9	SMAD3	L1FLnI_9q21.31k
ABR	MYO1E	CNKSR1	AMPD1	PARP15	CD96
POLH	BCL7A	AF127936.3	BMP6	TRIM22	NLRC5
BACH2	EBF1	TMEM39A	TLCD4	CR2	BANK1
MFHAS1	LCK	SPAG4	SCNN1B	SESN3	MCOLN2
GCSAM	PTK2	MCEE	ITGA8	EML4	TTC39C
DNASE1	IRAG2	IGHV4-61	BTD	CD47	CENPK
SGO1	LMO2	LINC02576	FXN	BANK1	DEK
NCAPD2	SORL1	RP11-114N1.1	FBXW7	L3MBTL3	MS4A1
CTB-193M12.5	CD72	PLPP5	MEI1	MGAT5	PIK3R6
HES6	HIVEP3	SSR4	ISCU	FMNL3	TMEM273
FANCD2	BCL6	GMPPA	MOXD1	CLEC17A	FCMR
TMPO-AS1	MCOLN2	TNFRSF17	ANKRD28	FCMR	CCDC141
TRIM59	IRF8	HML3_1q25.2	MTCYBP41	TRAF3IP3	ZBTB32
HAUS8	MS4A1	SIL1	TBCEL	DTX3L	NAPSB
DTX1	WEE1	SELENOM	RP11-720D4.2	KCNG1	SPIB
SLC6A6	LYSMD2	RUSF1	FER1L4	ABCB4	ITGAM
SLC9A7	PAX5	IGKV3-11	L1FLnI_5q21.3u	RP11-421L21.3	EPHA4
FAM241A	MARCKSL1	MLKL	HDLBP	PARP9	CDK5R1
RNF19B	ZC3H12D	CTD-2227E11.1	RNF207	RP11-429O22.1	WDR76
IL4R	BICDL1	FICD	KCNH2	GPR174	TRIM22
SSBP2	TMED8	MBNL2	FICD	L1FLnI_3q21.1c	CD1C
CRACD	TMOD2	PPP1R3B	CCPG1	PDE7B	CCR1
RCCD1	GCNT2	BET1L	CRELD2	LINC02397	TESPA1
CDK19	PIK3AP1	MTMR9LP	LMNA	VAV3	SYNPO
RUBCNL	DCK	DQX1	EPHA10	S1PR1	CDCA7
SLX4	ANKRD33B	NOL3	MINAR1	GAPT	FAM111B
IRF8	SYT11	CD79A	DENND2C	PPFIBP1	SIGLEC6
C21orf58	SYPL1	DENND6B	SEL1L	LPGAT1	BHLHE40-AS1
REXO5	GPR18	C1GALT1C1	CTSF	LINC00926	UST
RRM2B	HLA-DMB	IGKV3-15	PPFIA3	MYO7B	ZBED2
SGPP1	HTR3A	ATF6	REPS2	PLEKHA1	CSF1
ARHGEF39	GCNT1	SELENOS	RP11-44D5.1	TRAF5	GINS2
CRYBG1	CR2	BTG2	H1-10-AS1	STEAP1B	NCR3
JPT1	VNN2	STARD5	MDK	UST	L1FLnI_9q21.31i
KNSTRN	PANK1	MZB1	L1FLnI_10p13e	ABCB1	ITGAX
TACC3	CLEC17A	QPCTL	OS9	SH3BP2	WDHD1

RAD21	PUS10	FNDC3A	CCR2	MMP17	MYO1F
HMGN2	ARHGAP17	OSBPL3	CYFIP1	LAIR1	DHFR
TCL1A	ALOX5AP	IFNG-AS1	ACADVL	PCDH9	AHNAK2
APBB2	LHFPL2	TMC3-AS1	ASS1	C1orf162	TRAC
SUGCT	SLC16A2	ZBP1	NOL3	RP11-861A13.2	BRIP1
RBBP7	WDR76	ST6GAL1	SIL1	PARP14	CNR2
CIT	RGS8	ERN1	ZCCHC24	HPSE	SCML4
RASAL1	STAT6	IGKC	GRIK4	CRTC3	EPHB6
RACGAP1	MAML3	FBXW7	PDK1	FAM117B	TNR
MTA3	SERPINA9	PLD3	EIF2AK4	TMC8	CLNK
CKAP5	IL4R	LINC02352	INPP4A	DDX60L	BICDL1
RFTN1	P2RY12	CRELD2	GLDC	LBH	RP1-47M23.3
BCL6	BRI3BP	SERP1	CDC14B	SP110	H1-1
WEE1	PHF6	ACADVL	RAPGEF2	SHISAL2A	MCM4
CNTROB	PRAMENP	IGKV2-24	MB21D2	SCN3A	L1FLnI_9q21.31h
RMI2	MED12L	ARFGAP3	KIF13B	CEPT1	CCR7
BRI3BP	TMEM159	CFAP54	PLPP5	RASGRP2	NPAP1P4
KANK2	SPRED2	SLAMF7	LTK	FGR	TNFRSF1B
TMPO	FAM81A	FBXO16	SLC6A9	AC002480.4	CDC45
NDC1	LTA	APOL2	C11orf80	ITGA4	RP11-712B9.2
CFAP251	LAT2	EIF2AK4	BMI1	ARMH1	ERVLB4_14q23.3
TERF2	CD80	RWDD2A	LDLR	RP11-564A8.4	BHLHE40
CENPK	RP11-203B7.2	OGT	RIPOR3	SELL	CELF2-AS1
KIF22	SGPP1	TXNDC11	CALCOCO1	BTLA	CENPI
MCUB	SPIB	PPCDC	IGHV4-61	L1FLnI_6q13i	SELL
FAM83D	PHLPP1	ADA2	P3H3	DOP1B	PIK3R5
SLC35E3	RAPGEF5	AC026202.3	C12orf73	NIBAN3	FGR
TIMELESS	SH3RF1	IGHV4-28	SLC17A9	SPRY1	FAM81A
KDM1B	CENPK	IGLV3-21	L1FLnI_15q25.2a	L1FLnI_3q13.2a	FUT7
HMGB2	AFF2	MEI1	SAR1B	C12orf42	GPR34
ZNF831	CTD-2325P2.3	HERVP71A_8q24.13	TPM4	MACROD2	L1FLI_6p22.3
CENPI	RMI2	DENND2C	PLOD3	DENND11	MCM6
DEK	PAG1	SEL1L	PHLPP2	TGFBR2	E2F8
NCAPG2	HLA-DOA	ACP2	PLEKHN1	RBMS1	RMI2
GIHCG	NAPSB	IGLV1-47	RRAGD	MS4A1	XRCC2
PEX5	GALNT14	HM13	KIF19	HVCN1	ARAP2
AICDA	MYBL1	GMPPB	HOMER3	FCER2	GAPT

WDR76	TESPA1	GALK2	MYO5B	DNAH11	KYNU
UBE2C	BPNT1	CPEB4	CFLAR	ESAM	MCM2
PEG10	SAMD15	IGKV3-20	FNDC3A	MAML2	RRM2
CCNB2	CNR2	NKX6-3	ERN1	IFNGR1	MCM10
STMN1	SIGLEC10	CHPF	SLC41A2	ZBTB37	RP11-403N16.3
PTTG1	ACTN2	REXO2	MYO5C	RP11-564A8.8	SLC37A2
MYO1E	FEZ1	MANEA	IGHV3OR16-13	L1FLnI_5q11.2ta	ORC1
SMARCA4	L1FLnI_4q31.22d	ALG2	IGHV3OR16-8	P2RY14	NT5C3AP2
TROAP	FGD6	FER1L4	PPCDC	GCNT1	BRCA1
ZNF106	CAMK1	SAR1B	SLC38A4	GVINP1	EXO1
CENPO	LPP-AS2	SEC61A1	IGHV3OR16-17	SLC38A11	RAB31
LBR	SGO1	IGKV1-16	ARMC2	MTSS1	DSCC1
CDCA7	ASAP3	IGHV4-59	FAM13A	CHML	CDCA5
CKAP2	L1FLnI_14q32.13a	MINAR1	ABHD2	CD22	EML4-AS1
SLC2A5	BRIP1	CTD-2240J17.5	MYO1D	RP11-452F19.4	LTB
PSRC1	IL7	IGHV1-18	RPS27AP8	SNX18	CAPG
LINC01991	ZNF608	LINC00698	SELENOM	CR1	AC008697.1
PTK2	LINC02099	TMEM214	FBH1	CELF2-AS1	L1CAM
EZR	DEF8	CCPG1	ITPRIP	CNR2	H2BC13
KIF20A	FCRL3	FKBP11	PSAP	ZNF528-AS1	ASF1B
CCNB1	LPP	FBXL8	PIP5KL1	GBP4	CTD-2509G16.5
SPDL1	DMD	CHPF2	C16orf54	MARCHF1	RASGRP2
PAX5	HOPX	L1FLnI_3q23t	RAB3D	IL24	ESCO2
HMGN2P46	CDCA7	RP1-134E15.3	IGHV3OR16-9	HML2_1q22	ADAMTS6
SLC30A4	VAV3	ARMCX3	GOLGA2	RP11-281P23.3	H2BC9
STIL	REL	PDIK1L	RAB36	FAM177B	TK1
NUF2	WDHD1	ACOXL	LINC02711	SP100	LINC01991
TPX2	DEK	GLRX	VPS37B	CTNND1	GPR82
CKS1B	MAST2	HERVP71A_3p26.1	CSGALNACT1	PAXIP1-AS2	TNFSF12
MYBL1	U62631.5	DERL3	FAM114A1	CD1A	LFNG
DBF4B	B3GALNT1	ARF4	ATP8B2	RP3-323N1.2	CD80
CTPS2	FCRLA	FNDC3B	FNDC3B	LIX1-AS1	POC1A
DNMT3B	HLA-DRA	EDEM2	LYPD6B	CASP4LP	AICDA
KIF18B	L1FLnI_15q22.2a	MINDY1	BMP8B	ZNF528	CCNA2
POU4F1	CAMK2B	PGM3	TTLL7	ZBTB16	CCNB1

ARL6IP1	KCNMB4	RP11-294C11.2	PERP	SATB1-AS1	CR1
ESPL1	EPS15-AS1	IGKV1-12	NPC2	DDX60	CCR5
FOXM1	CDK5R1	RPN2	IGF1	ARHGAP15	OSTN-AS1
C1orf112	SNX29P1	IGHV3-21	TXNDC11	JAM3	RP11-403N16.4
SYBU	HELLS	NXPE3	CTD-2653D5.1	CD1C	CXCR3
FBXO43	SLC1A1	RAB3D	CLPTM1L	BEND5	TTC24
ERCC6L	RP13-786C16.1	MIR5571	PECAM1	ABAT	DTL
WDR62	RP11-231C14.7	IGHV3-30	RAPGEF3	DEXI	TNFRSF13B
NDC80	AC023590.1	XBP1	RDX	MX2	CLSPN
DEPDC1B	AC073043.1	IGHV4-39	HEXB	HHEX	TGM2
NEIL1	BCL2A1	METTL7A	GPR176	ZCCHC18	RP3-323N1.2
TRAF5	L1FLnI_4q13.3j	AFF1	CASP10	AC104530.1	AHNAK
ALPK1	LINC01991	LARP1B	PRDX4	RHOBTB1	CIITA
CDKN3	RP11-415F23.5	L1FLnI_5q33.3l	FAM174A	HS3ST1	PREX1
AURKB	LRRC32	PLEKHN1	UAP1	CCDC141	SCIMP
CENPN	TCL1A	IGHJ5	HSP90B1	PRICKLE1	PCLAF
DNMT1	TMEM229B	C1R	RP11-665E10.2	L1FLnI_1q32.2e	DDX60L
CENPH	CFAP20DC	TXNDC15	ARID3B	PEAK1	CDC20
KIFC1	PXDN	PRDX4	TMEM59	ARAP2	CALHM2
BCAS4	RPRD1B	CLIP4	RBM47	L1FLnI_4q32.2i	DMC1
KIF2C	LDHAL6B	ZCCHC24	CD63	TPK1	E2F1
GTSE1	NLRP4	JCHAIN	SELENOS	L1FLnI_11q12.1v	AURKB
RAD51AP1	ANKLE1	IGKV4-1	ELL2	MOB3B	H2BC10
CENPL	LINC02137	SRPRB	FKBP11	EML4-AS1	NPAP1P6
CDC25C	ADARB1	ERVLE_2p14b	WDR45	RP11-35G9.3	RIN3
AFF2	GPR137B	TMC3	SLAMF7	PTPRK	PLAC4
CCNA2	DLGAP1	LINC02227	WIPI1	IFNK	ZWINT
CDK1	PCDHGC4	RBM47	TCN2	P2RY10	CD247
CDCA2	RP11-131H24.4	LINC01485	FGFRL1	BCL11A	KIFC1
NEIL3	UGT8	PREB	UBE2QL1	GPR65	SERPINB6
DHFR	KIF5C	IL10RA	JSRP1	HLA-DMB	H1-5
HJURP	SCIMP	IGHV3OR16-9	SCAMP5	BTBD6P1	H4C13
FAM81A	CIITA	PIM2	B9D1	TTC24	PBK
NSD2	E2F8	PDIA4	ZNF275	HLA-DPB1	CCL22
DCP2	EBI3	IGKV3D-11	SEPTIN10	HLA-DOA	STMN1
TTK	LINC01857	RP11-490O6.2	BCL2	ST3GAL1	CHAF1B

IQGAP3	RGS13	B9D1	TMEM63B	C8orf37	ACP5
HERV3_14q32.33	HERVI_15q25.1	HML3_1q25.2	HML2_20q11.22	HML2_1q22	ERVLB4_14q23.3
HERVFH21_1p36.31	ERVLB4_6p23	HERVP71A_8q24.13	MER4_9p13.3a	HERVL_2p12a	HERVL_2p12a
MER61_3q13.11	ERVLB4_8p21.3b	HERVP71A_3p26.1	MER4_9p13.3b	HERVFRD_2p12a	MER61_3q13.11
HERVFH21_7q11.21	MER101_2p25.2	HML2_1q23.3	HERVP71A_15q24.2	HERV9_6q25.1	HARLEQUIN_5q33.3
HARLEQUIN_19p12b	HERVH_2p14b	HERVP71A_15q24.2	ERV316A3_15q25.2a	HERVK11_1q23.3a	ERVLE_9q21.31d
HERVL_1q23.3a	HERV3_14q32.33	HML3_8q24.13	ERV316A3_5q15a	PRIMA41_Yq11.223a	MER61_19p12c
MER4_2q14.1b	ERV316A3_2q35i	ERVLB4_15q13.3a	ERVLE_4q31.3a	HML5_Yq11.223e	HERVFRD_2p12a
HERVL_12p13.1b	MER41_4q31.1	ERVLE_2p14b	HERVW_2q24.3	HUERSP2_6p22.3	HARLEQUIN_19p12b
ERV316A3_Xp21.2	HERVL_12p13.1b	LTR25_3q24a	HERVL18_7q31.32	MER4B_20q13.12	HML2_1q22
HML5_12q23.1	HML5_3q26.2	HERVL40_11q13.4b	MER101_19q13.2c	PRIMA41_Yq11.223b	PABLB_7q11.21
HERVL66_19p12f	HERVE_11q13.4c	HARLEQUIN_11q13.4	HERVH_9p13.3b	ERV316A3_6q24.1a	HERVL_5q12.3
HML5_Xq11.2a	HUERSP2_22q11.22	MER4_22q12.3	HERVP71A_8q24.13	HERVIP10FH_2p14	HERVH_9q21.31a
ERVLB4_8p21.3b	HERVL18_11q22.3	HERVL40_19q13.33	HERVL18_11q14.2b	ERVLB4_6p23	MER4_19p12a
MER61_19p12c	HERVL_3q13.11c	HUERSP2_19q13.2	ERV316A3_17q24.3a	HERVK11_1q23.3b	ERV316A3_12q24.13
HERVIP10F_11q24.2	MER4_19p12a	HERVFH21_Xq11.2a	ERVLE_5q21.3b	ERVLE_5q31.1a	MER4_2p11.2a
HERVIP10F_2q21.2	MER61_19p12c	ERV316A3_3q24a	ERV316A3_8q13.3a	HERVH_19p13.2a	HERVL18_1q32.2
MER101_3q26.31	HERVEA_6q22.31	ERVLB4_16q13b	PABLB_2q31.1	MER4_7q21.12	HERVH_12q13.2b
MER4_19p12a	HERVL_17p11.2b	MER61_3q24a	HERVL_6p25.2	ERVL_Xq21.1a	ERVLE_8q21.13g
HERV3_1q23.3	HML2_4p16.3a	ERVLE_16p11.2b	HERV9_10p13	HERV3_16p13.3	MER34B_1q23.3b
ERV316A3_1p34.3c	MER61_3q13.11	HERVFH21_Xq11.2b	HML6_14q24.2	ERV316A3_12q24.13	HERVL_12p13.1b
HERVIP10FH_17q21.32	MER101_2p16.3	ERVLB4_3q12.3	ERVLE_18q11.2c	MER4_2p11.2a	HERV3_19p13.3
MER4_8p11.1b	HUERSP2_7q35	HERV9_10p13	ERV316A3_2p25.1a	ERV316A3_10q23.33	MER41_1q44a
ERVLB4_3q25.2c	HARLEQUIN_10q23.1	PABLB_2q31.1	ERVLE_8q24.22g	ERVLE_8q21.13g	ERVLE_15q15.1
ERVL_6q15	HERVL18_1q32.2	MER61_12q13.12	HML1_6q23.2	ERVLE_4q21.23a	HERV3_4p16.1
HUERSP3_8p11.1b	HARLEQUIN_19p12b	HERVIP10FH_19q13.43	HERVP71A_2q32.2	ERV316A3_21q21.2g	ERVLE_4q21.23a

Supplementary Table 3: Top genes and HERVs from the B-AG dataset used to create B-cell-specific sets for HAGSEAS analysis
